# Characterization of colonization kinetics and virulence potential of *Salmonella* Enteritidis in chickens by photonic detection

**DOI:** 10.3389/fvets.2022.948448

**Published:** 2022-08-02

**Authors:** Dinesh H. Wellawa, Po-King S. Lam, Aaron P. White, Brenda Allan, Wolfgang Köster

**Affiliations:** ^1^Vaccine and Infectious Disease Organization, University of Saskatchewan, Saskatoon, SK, Canada; ^2^Department of Veterinary Microbiology, Western College of Veterinary Medicine, University of Saskatchewan, Saskatoon, SK, Canada; ^3^Department of Veterinary Pathology, Western College of Veterinary Medicine, University of Saskatchewan, Saskatoon, SK, Canada

**Keywords:** *Salmonella*, bioluminescence, lumazine, virulence, yolk sac infection, SPI-1, *fur* and *tonB*

## Abstract

The light emitting module *lux* operon (*luxCDABE*) of *Photorhabdus luminescens* can be integrated into a “dark” bacterium for expression under a suitable promoter. The technique has been used to monitor kinetics of infection, e.g., by studying gene expression in *Salmonella* using mouse models *in vivo* and *ex vivo*. Here, we applied the bioluminescence imaging (BLI) technique to track *Salmonella* Enteritidis (SEn) strains carrying the *lux* operon expressed under a constitutive promoter sequence (sigma 70) in chicken after oral challenge. Detectable photon signals were localized in the crop, small intestine, cecum, and yolk sac in orally gavaged birds. The level of colonization was determined by quantification of signal intensity and SEn prevalence in the cecum and yolk sac. Furthermore, an isogenic SEn mutant strain tagged with the lux operon allowed for us to assess virulence determinants regarding their role in colonization of the cecum and yolk sac. Interestingly, mutations of *SPI-1*(Salmonella Pathogenicity Island 1) and *fur* (ferric uptake regulator) showed significantly decreased colonization in yolk sac that was correlated with the BLI data. A similar trend was detected in a Δ*tonB* strain by analyzing enrichment culture data. The inherently low quantum yield, light scattering, and absorption by tissues did not facilitate detection of signals from live birds. However, the detection limit of *lux* operon has the potential to be improved by resonance energy transfer to a secondary molecule. As a proof-of-concept, we were able to show that sensitization of a fluorescent-bound molecule known as the lumazine protein (LumP) improved the limit of detection to a certain extent.

## Introduction

**Figure 1:**
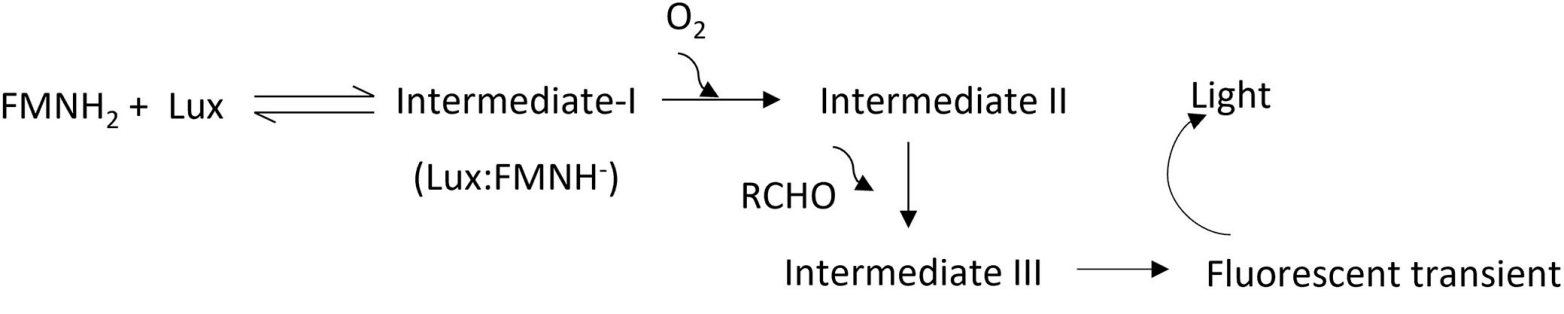
Proposed light-emitting reaction. Intermediates; I, II, and III are produced during the engagement of bacterial luciferase (Lux) with flavin, oxygen, and aldehydes. A florescent transient is produced during the degradation of intermediates and believed to be excited by other high-energy intermediates during degradation of intermediate III. FMNH_2_: reduced riboflavin phosphate, and RCHO: aliphatic aldehyde.

Photons emitted during a bioluminescent reaction can be captured with a cooled charged coupled device (CCD) camera and converted into an electron charge. The recorded electrical charge pattern is then utilized to generate an image. This is the basis of a bioluminescent imaging (BLI) system to detect the light source generated within a tissue. The light-emitting module of bacterial origin, *luxCDABE*, encodes all enzymes needed to produce signals and is advantageous because no reagents are needed to be injected during imaging (1). Also, the expression of the *lux* operon allows for monitoring of the kinetics of most prokaryotic infection processes. The light-emitting reaction is catalyzed by the enzyme luciferase encoded by *luxAB*. In the presence of molecular oxygen, luciferase catalyzes the oxidation of a long chain aldehyde (luciferin) into an aliphatic carboxylic acid using FMNH_2_ (reduced flavin mononucleotide) as a cofactor (2). During the oxidation process, substrates form excited intermediate compounds and release photons in a broad spectrum (490 nm maximum) with a low quantum yield (<1; Figure 1) (3). The key substrate in the reaction, aliphatic aldehyde, is supplied and recycled in the bioluminescent reaction by fatty acid reductase systems encoded by *luxCDE*. FMNH_2_, is considered as abundant since bacteria encodes for *de novo* synthesis of riboflavin (vitamin B2), which is the precursor for flavin compounds (4). Hence, the rate-limiting step of the light emitting reaction catalyzed by bacterial luciferase is molecular oxygen. The first detailed feasibility of BLI to track bacterial infection was described by Contag et al. (5) using mice infected with *Salmonella* Typhimurium (STm) strains expressing the *lux* operon from *Photorhabdus luminescens*. Live imaging of mice up to 8 days revealed that the bioluminescent signal was localized in the cecum, suggesting that STm persistently infected the cecum after oral gavage. Furthermore, the study revealed that oxygen is a limiting factor to generate bioluminescence in the gastrointestinal environment because it is more anaerobic (5). To our knowledge, a similar kinetic analysis using *Salmonella* serovars during gastrointestinal infection in chicken has not been published.

Chicken (*Gallus gallus domesticus*) is a reservoir host for non-typhoidal *Salmonella* (NTS) *s*erovars and responsible for most food-borne human salmonellosis globally (6). The *Salmonella enterica* subspecies *enteric* serovar Enteritidis (SEn) has become one of the most dominant serovars in poultry (different phage type exists) on most continents including America, Europe, Africa, and Asia. There are number of genetic traits of SEn governed by pseudogenization of metabolic pathways, acquisition of pathogenicity islands, virulence plasmids, invasins, fimbria, and acid tolerance response thus leading to a broad host range of SEn (7). However, it is not fully understood how each of these genes contribute to efficient colonization in chicken. In a report published by Li et al. (8), it was shown that the global spread of SEn is critically linked to breeders' harboring of SEn, reinstating that our understanding of kinetics of colonization, harborage sites, and colonization mechanisms remains largely unknown regarding the reservoir avian host. In this sense, we believe that the development of a BLI in a chicken model will be valuable in refining colonization niches and understanding the colonization kinetics of SEn, which is not accurately determined through conventional methods (9, 10). For example, random sampling of tissue may miss out on important colonization sites. Once established, a working BLI model can be used for gene expression studies to facilitate-high throughput screening of virulence factors in SEn.

This study describes our efforts to analyze early kinetic events of SEn infection in day-old chicken by BLI. The detection limit of the current reporter strength was determined regarding several colonization sites. Furthermore, we have described a way to improve the signal intensity of the current reporter by allowing for energy transfer to an accessory protein, which possesses a florescent molecule. The improved reporter will be advantageous in future in gene expression studies. During the study, we were able to visualize tissue localization of various mutant strains of SEn tagged with the *lux* operon, thus enabling us to assess for virulence determinants that are not well characterized in chicken.

## Results

### *In vitro* characterization of bioluminescence signal strength and growth of *S*. Enteritidis carrying the bioluminescent reporter

**Figure 2:**
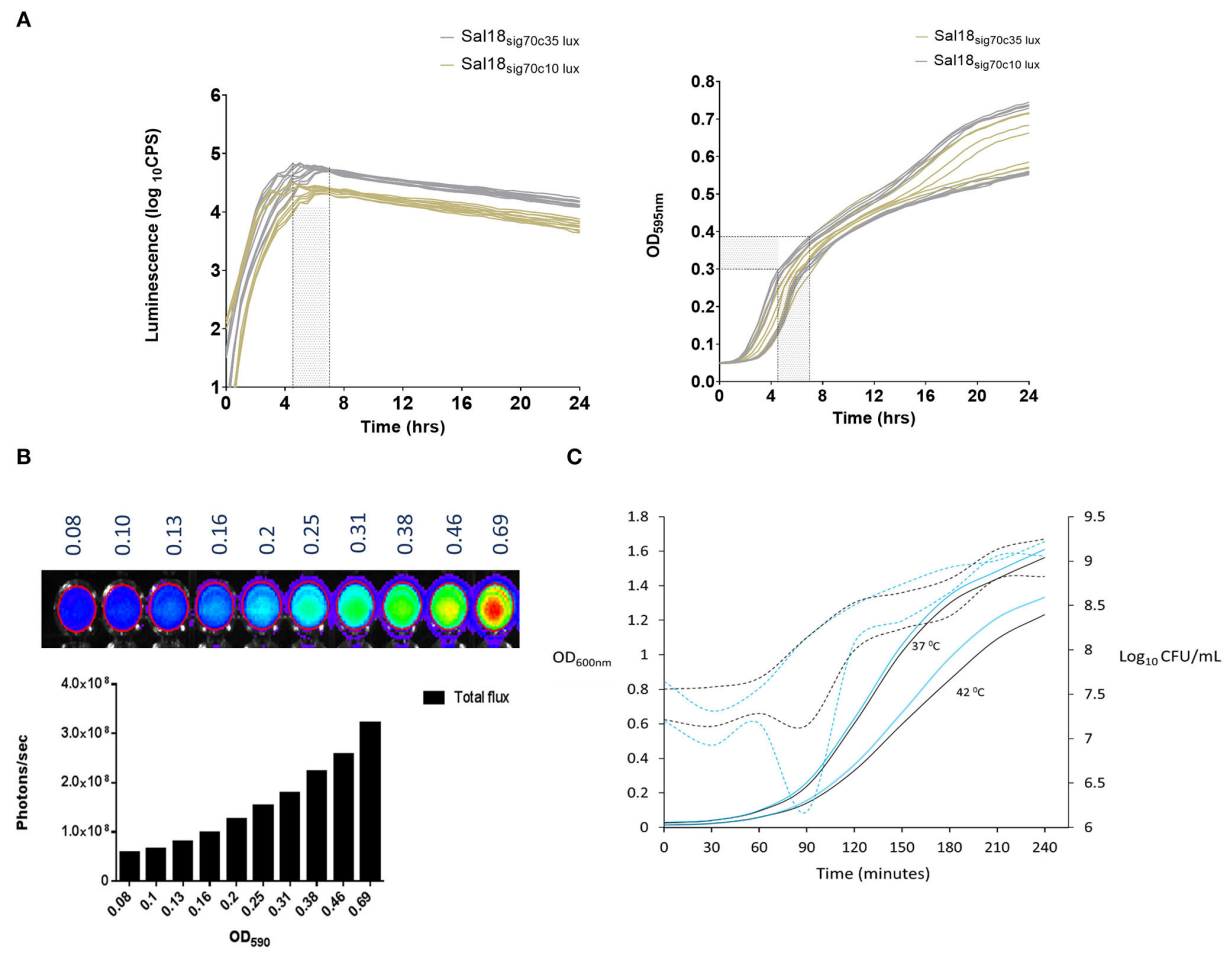
*In vitro* kinetics of luminescence expression and growth of *Salmonella* Enteritidis expressed under a constitutive promoter. **(A)** Level of *lux* operon expression and growth under two different promoters over time. Lux operon expression was measured as means of light signal production. Luciferase assay was performed using a Victor V^3^ plate reader (Perkin Elmer). Strains: Sal18_sig70c10lux_ and Sal18_sig70c35lux_. **(B)** Correlation between cell densities and light emission of Sal18_sig70c35lux._. Culture with OD_600nm_ of 0.7 was diluted at 1:1.25 with an LB medium. Red circle marks the demarcation for total flux calculation with the software. Total flux: cumulative scores of photons/second recorded by each pixel in the CCD camera of the whole animal imager (IVIS Lumina II). Setting of the IVIS Lumina II; exposure: 3 sec, f = 1, binning = 4. **(C)** Growth curve of the Sal18 wild-type strain and Sal18_sig70c35lux_ grown in the LB medium (1:100). Overnight cultures were obtained from SEn strains grown in the LB medium at 37°C. Solid lines represent OD and dotted lines CFU/ml. The experiment was performed once Black line: wild-type Sal 18, blue line: Sal18_sig70c35lux_.

We incorporated the *lux* operon of *Photorhabdus luminescens* into the *Salmonella* Enteritidis chromosome using the modular Tn7-based systems described by Shivak et al. (11). In this methodology, the *lux* operon carried in a miniTn7 vector flanked by Tn7 elements (right and left) was inserted into the attTn7 site in the SEn chromosome. This was achieved by transposases (*tnsABCD*) carried by helper plasmid pHSG415 (11). Shivak et al. constructed two strengths of the synthetic promoter derived from a σ^70^-dependent consensus sequence: sig70c35 and sig70c10. Sigma 70 factors are essential proteins that direct DNA-dependent RNA polymerases to designated transcription start sites to constitutively express “housekeeping” genes in a bacterial cell during growth (12). The consensus sequence TTGACA in 35 base pairs upstream from the transcription start site of the double stranded DNA (-35) showed high expression of the *lux* operon (sig70c35 lux) and increased light production in *Salmonella* Typhimurium (STm) (11). The promoter containing sequence TATAA in the−10 region (sig70c10) was commuted to a more moderate level of expression than sig70c35 (11). In line with that, SEn chicken isolate Sal18 showed a mean maximum of ~64,000 counts per second (CPS) of luminescence after being transformed with the sig70c35 *lux* (Sal18_sig70c35lux_) while being transformed with the sig70c10 *lux* gave a maximum of ~26,000 CPS as determined with a plate reader (Figure 2A). Maximum signal intensities of both reporters were achieved between OD_595nm_ 0.3 and 0.4 (shaded areas in Figure 2A). SEn continued to grow beyond OD 0.4 in the liquid culture (145 μl), but the generated bioluminescent signal declined at a steady rate over time (Figure 2A). We were able to measure the total emitted light signals (photons/s) from a 200-μl aliquot of Sal18_sig70c35lux_ as detected with the CCD camera in the whole animal imager (IVIS Lumina II; Figure 2B). The Sal18_sig70c35lux_ was grown to an OD_600nm_ ~0.7 in a liquid culture (50 ml) and diluted to give rise to varied cell densities determined by OD values (Figure 2B). An undiluted sample (OD = 0.69) emitted ~3 ×10^8^ photons/s and then gradually decreased at each dilution (1:1.25) to a level of <10^8^ photons/s after the sixth dilution (OD ~0.16). This indicated that emission of a bioluminescence signal from a cell population of a SEn strain in the same exponential growth phase is positively correlated with bacterial cell densities (*R*^2^ = 0.9764). Furthermore, the expression of the *lux* operon under the sig70c35 promoter did not alter the growth rate compared to its wild type in a nutrient rich medium (Figure 2C). The overnight culture of strain Sal18 grown at 37°C showed a longer lag phase getting into the exponential growth phase after subculturing at 42°C (Figure 2C). Even though there was an initial lag phase, at 4 h the SEn strains grown at 42°C reached a cell density of ~10^9^ CFU/ml, which is similar to cells grown at 37°C (Figure 2C). The body temperature of a newly hatched chicken is around ~39.7°C, and in adult birds it is ~42°C, which is higher than in most mammals. Our growth curve analysis suggested that SEn strains carrying bioluminescent reporters will be able to grow in similar rate as the wild type of strain at temperatures in the bird. The growth patterns of the reporter was further confirmed by assaying multiple times using a plate reader (Victor X^3^-Perkin Elmer), which did not show a defect at 37°C or switching to 42°C (Supplementary Figure 1).

### Constitutive expression of the *lux* operon under the promoter sig70c35 facilitates detection of early kinetic events during gastrointestinal colonization of *S*. Enteritidis in chicken (animal experiment 1)

**Figure 3:**
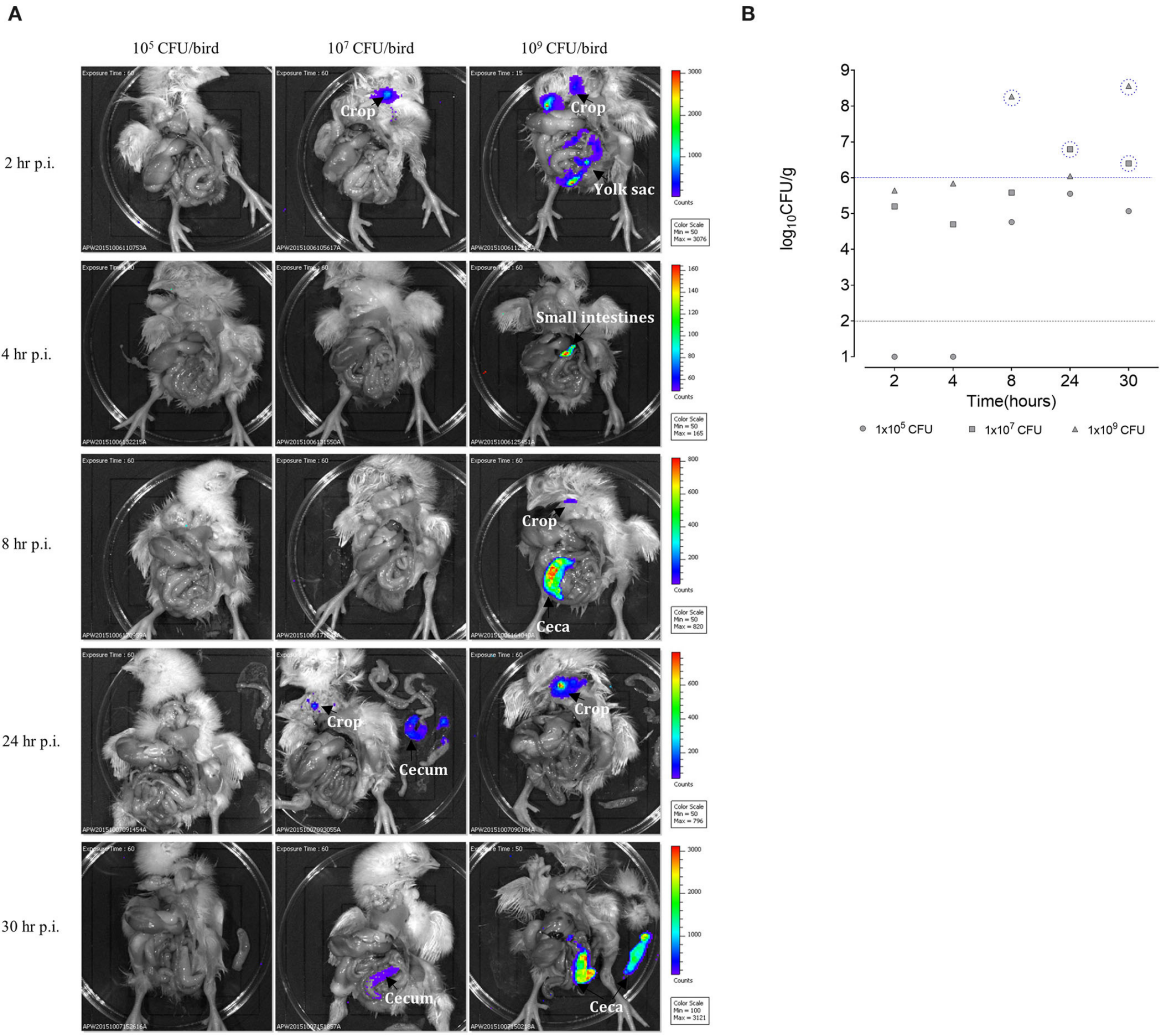
**(A)** *Ex vivo* imaging of the day-old SPF birds challenged with SEn carrying the sig70c35 lux reporter. Each color-coded area represents a photon captured with the CCD camera in counts. Setting of the IVIS Lumina II: auto exposure: *f* = 1, binning: 8. Background bioluminescent was set at 50 counts as determined by images taken from unchallenged birds (data not shown). **(B)** Bacterial load in the cecum. Dilutions were made from the cecal content enumerated in brilliant green agar plates. Each symbol represents a bird. Black dotted line is the detection limit of the brilliant green agar plate. Blue dotted line indicates the limit of detection of IVIS Lumina II in visualizing the SEn in the cecum under settings described here.

Three groups of SPF birds (five birds/group) were orally gavaged with the Sal18_sig70c35lux_ reporter. Each group was challenged with a different number of bacteria: 10^5^, 10^7^, and, 10^9^ CFU per bird. At each time point, a single bird was euthanized and imaged using the an animal imager (IVIS Lumia II) to track the SEn strain in the gastrointestinal tract. The birds orally challenged with the 10^5^ CFU dose did not reveal detectable bioluminescent signals from the gastrointestinal tract at all time points examined (Figure 3A). The birds challenged with the 10^7^ CFU dose gave a detectable signal from the crop region 2 h post infection (p.i.), with intensities ranging from 50 to 700 counts after 60 s of exposure time. Signal intensities with similar range of photon counts were detected from the crop of a chick challenged with 10^9^ CFU at 2 h p.i. (Figure 3A) during a 15-s exposure. Our data suggested that a portion of the challenged SEn remained in the crop region before “traveling” to the lower part of the GI tract. Furthermore, strong bioluminescent signals were detected in the small intestine of the birds challenged with 10^9^ CFU at 2 h p.i.. Some signal intensities reached >1,000 counts (Figure 3A, 10^9^ CFU dose) in the small intestine, reflecting that a major population of SEn may reside in the small intestine at 2 h p.i. However, we did not quantify the intestinal bacterial burden in different locations as directed by imaging. The estimated *Salmonella* burden in the cecum at 2 h p.i., was ~10^5^ CFU/g from the birds infected with high challenge doses (10^7^ and 10^9^ CFU; Figure 3B). Reporter strains at a concentration of ~10^5^ CFU/g of the cecum did not give detectable signals (15- or 60-s exposure); hence, we believe that to be detected from the intestinal tract, the reporter strain needs to reach a population of >10^5^ CFU/g in 1-day old birds.

A major bioluminescent signal detected at 4 h p.i. was from the small intestine (ileum region) of the birds challenged with 10^9^CFU with intensities ranging ~100–~140 photon counts. This range of signal intensity was lower than what we observed from the small intestine at 2 h p.i. (> 1,000 counts) in the same group. The low-signal intensity in the small intestine may be correlated with the low SEn concentration at 4 h p.i. compared to 2 h p.i. Since the small intestine has regular peristaltic movements, it is plausible that SEn will not remain in the intestinal lumen in high numbers. The cecal bacterial load remained ~10^5^ CFU/g in the birds challenged with 10^9^ CFU and did not result in a bioluminescent signal, confirming that only a population of >10^5^ CFU/g can be detected with the bioluminescent imager (Figure 3B). The bird challenged with the 10^7^ CFU oral dose contained ~10^4^ CFU/g at 4 h p.i. (Figure 3B). It was relatively lower than the quantified bacterial load at 2 h p.i. in the same group (Figure 3B). The lowest dose of 10^5^ CFU did not result in recovery of SEn from the cecum at this time point, which is similar to 2 h p.i.

The birds that obtained the highest oral dose of SEn emitted signals from the ceca with surface areas consisting of 200–800 photon counts at 8-h p.i (Figure 3A). The emission corresponded to a population of ~10^8^ CFU/g of SEn as estimated by cecal material processing and plating on selective media (Figure 3B). In parallel, the birds infected with the 10^5^ CFU oral dose reached ~10^4^ CFU/g, and the birds infected with 10^7^ CFU had ~10^5^ CFU/g (Figure 3B) with no signal emissions. In chickens, the food material takes 6 h to travel into the cecum after ingestion. Hence, we expected a relatively high load of SEn in the cecal material at 8 h p.i. In line with this, we observed a dose-dependent seeding of the cecum reaching a comparatively high concentration from the birds challenged with each dose. In addition to the cecum, a bioluminescent signal was detected from the crop at 8 h p.i. in the birds challenged with 10^9^ CFU (Figure 3B). It had ~50 counts of surface intensities, which were relatively low compared to the signal generated at 2-h p.i (200–400 photon counts). We speculate that low bacterial concentration in the crop may account for the low-signal intensity at 8-h p.i compared to 2 h p.i. Furthermore, the imaging data suggested that the majority of the challenge doses had migrated into the intestines 8 h p.i. Even though we did not quantify the bacterial burden in the crop content, the emission of bioluminescent signals from the crop at early time points suggested that the crop may act as a transient attachment/colonization site for SEn.

Digested food materials can be retained up to 12 h in the chicken cecum before emptying into the large intestine for expulsion as a fecal material (13). In nature, this is not a fully synchronized process and greatly varies among individual birds. We saw a dramatic drop in SEn burden in the cecum from ~10^8^ (1.85 ×10^8^) to ~10^6^ CFU/g (1.11 ×10^6^) at 24 h p.i. in the birds challenged with the highest oral dose (Figure 3B). We suggest that this drop in *Salmonella* burden resulted from the emptying of cecal materials into the large intestine that occurred during the 24-h infection window. At this concentration, there were no signals detected from the cecum, while the bird challenged with 10^7^ CFU dose gave a detectable signal at SEn concentration of ~6 ×10^6^ CFU/g cecal material. Taken together, we concluded that the detection limit of SEn by *ex vivo* imaging (IVIS Lumina II) was ~1 ×10^6^CFU/g using the current reporter strength on exposure at 60 s. The crop material from the birds challenged with the 10^9^ CFU oral dose had bioluminescent signal intensities reaching a maximum of 800 photon counts at 24 h p.i. but there was no signal from the cecum. In parallel, the birds challenged with the 10^7^ CFU oral dose also had a small area of intensity reaching 100 photon counts in the crop material. It is plausible that during the 24-h infection window SEn has been shed into the environment in adequate amount and then was transmitted by contamination of feed or water. Hence, a signal generated from the crop materials at 24 h p.i. was highly suggestive of SEn transmission from the environment to the birds. In the future, transmission events of *Salmonella* may be accurately tracked by simply imaging crop materials rather than randomly collecting environmental samples. The crop acts as a transient storage diverticulum, since such material can be found at frequent time points at ease for this purpose. The imaging data confirmed that during the next 6 h (30 p.i.) the crop was completely emptied (less material), which suggested that anything which was stored had been moved to the intestines (Figure 3A). At 30 h p.i., in birds other than those challenged with the lowest dose, SEn reached a cecal concentration of more than the detection limit (Figure 3B), resulting in bioluminescent signals from the cecum (Figure 3A). The SEn burden of ~ 4 ×10^8^ CFU/g of cecal material resulted in signal intensities ranging from 1,000 to 3,000 photon counts at 30 h p.i. (10^9^ dose), which was the highest intensity so far from the cecum (Figure 3B). In parallel, ~2.5 ×10^6^ CFU/g of an SEn population gave rise to 100 photon counts (10^7^ dose, Figure 3A). The difference between the two signal intensities can partly correspond to the bacterial concentration at this time point. However, when the signal intensities emitted from the cecum at 8 h p.i. were compared to 30 h p.i. of the birds challenged with the highest oral dose, surface signal intensities from the cecum significantly differed even though both times shared a similar SEn burden (~10^8^ CFU/g). The signal generated at 30 h p.i. (1,000–3,000 counts) was significantly higher than the 8-h p.i. (200–800; Figure 3B). We suggest that this will be largely related to the relatively high transcription of the *lux* operon by the SEn strain residing in the cecum milieu at 30 h p.i. compared to 8 h p.i. As mentioned earlier, the expression of the *lux* operon is a driven by a sigma 70 factor-recognized promoter, which expressed constitutively to activate housekeeping factors inside the bacterium. Hence, we suggest that this gain in signal intensities marks a true colonization event taking place by SEn with chicken's cecum milieu at 30 h p.i. We hypothesized that SEn actively produced more sigma 70 factors to keep up with the metabolically demanding process in establishing the cecal environment. Overall, our imaging data together with colony counts suggested that SEn actively begins to colonize the cecum 30-h post infection, and that this is a dose-dependent process. Also, we conclude that a normal dose of 10^9^ CFU per bird is an optimal challenge dose for visualizing early kinetic events using SEn strains carrying the lux operon with current signal strength.

### Lumazine protein from *Photobacterium leiognathi* increases the signal intensity generated by the *lux* operon of *Photorhabdus luminescens*

**Figure 4:**
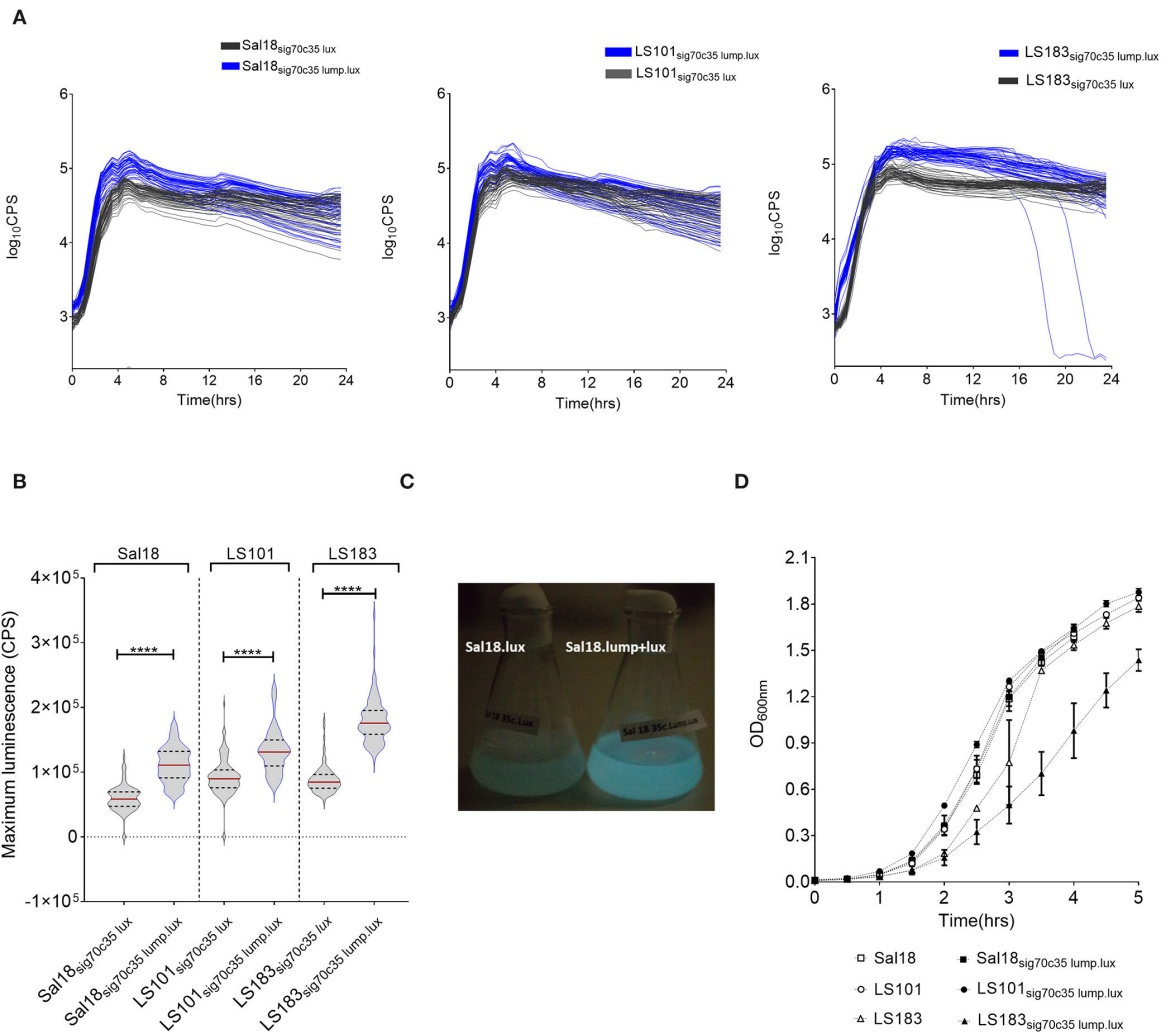
Modification in the light signal produced by different poultry isolates of *Salmonella* Enteritidis by LumP (lumazine protein). **(A)** Kinetics of light production by *S*. Enteritidis expressing luciferase and the lumP. An assay was performed using a Victor V3 plate reader on bacteria grown at 37°C. Each line indicates one biological replicate, and 48 biological replicates were represented here. **(B)** Violin plot of maximum light signal generated by each biological replicate presented in **(A)**. The horizontal bar represents the median value while the upper and lower dashed lines are indicative of the 3rd and 1st quartiles, respectively. Median values were statistically analyzed. ^****^p = <0.0001. **(C)** Photographs of light emission from cultures of S. Enteritidis isolate Sal18. Overnight cultures were subculture at 1:100 in the LB medium to an OD_600nm_ value of 8. **(D)** Growth curves grown in LB media. Each symbol represents mean with standard deviation of the optical density measured at 600 nm. The assay was run thrice using one biological replicate at each run.

**Figure 5:**
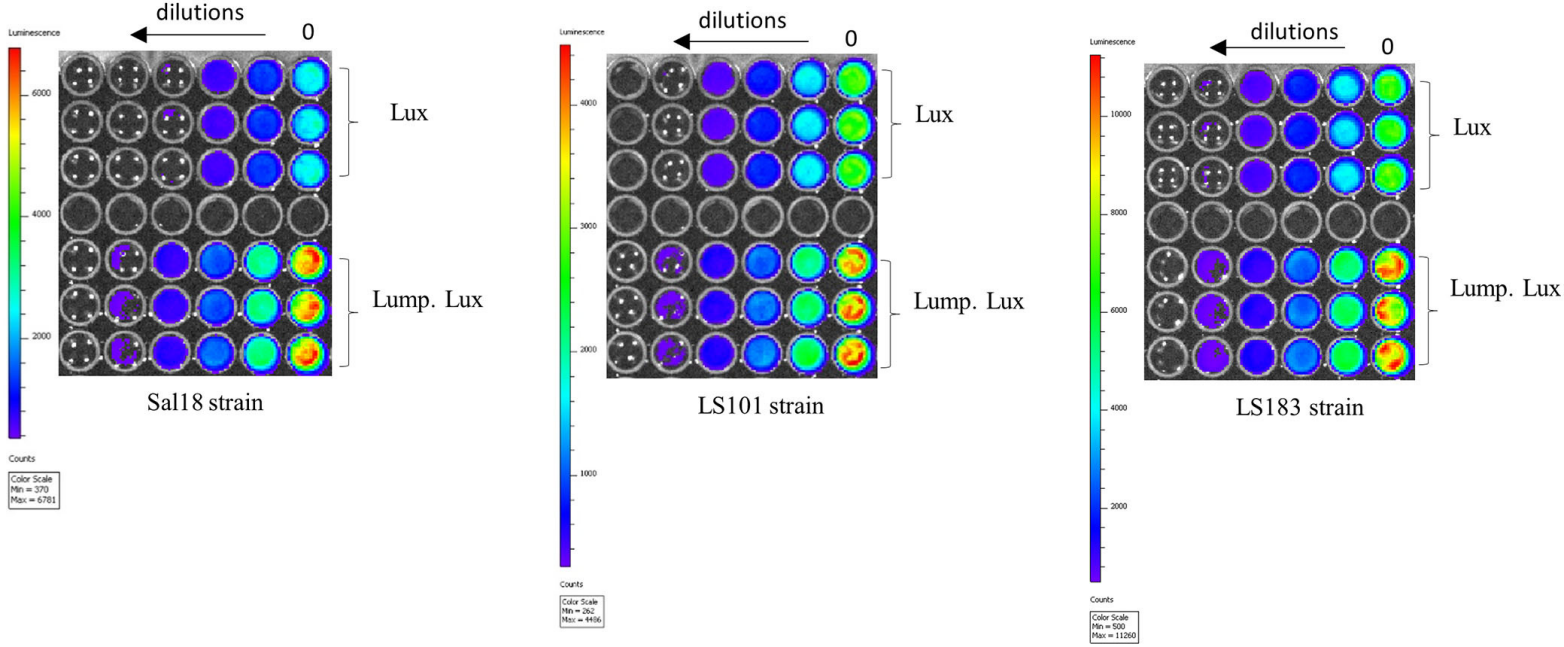
*In vitro* detection limit of various SEn strains carrying the reporter. Each strain was grown to an OD_600nm_ of 0.7 (dilution 0) and diluted (two-fold**)**. Aliquots 200 μl were placed on a 96-well plate. IVIS Lumina II setting: *f* = 1, expo = 5 s, and bin = medium.

We hypothesized that the lumazine protein from *Photobacterium leiognathi* will closely interact with the luciferase enzyme from *Photorhabdus luminescens* and will give a brighter intensity of light that potentially could improve the detection limit. The lumazine protein (LumP) possesses a non-covalently bound fluorophore, 6,7-dimethyl-8-ribityllumazine (DMRL), which interacts closely with the alpha subunit of the luciferase enzyme (14, 15). This fluorophore acts as a secondary emitter of the *Photobacterium* species that generates brighter blue light (475 nm) in these bacteria. In our construct, *lump* was cloned upstream of the *lux* operon of *Photorhabdus luminescens* and expressed under the sigma 70c35 promoter (sig70c35 lump lux). The measured light production over time by SEn strains expressing the sig70c35 lump lux showed relatively faster kinetics (blue line in Figure 4A) and reached a new maximum compared to the original construct (Figure 4B). Calculated relative fold changes in the maximum luminescence signal for Sal18, LS101, and LS183 were 1.9, 1.4, and 2, respectively (relative fold change = mean max *lumP* reporter/mean max *lux* reporter; Figure 4B). The bright blue light was visualized from the Sal18_sig70c35lumplux_ culture grown to OD_600_ = 0.7 compared to Sal18_sig70c35lux_ (blue to green color), indicating that bioluminescence has shifted to blue color (Figure 4C). LS183_sig70c35lumplux_ showed the highest light intensity determined with the plate reader in a 145-μl volume culture reaching ~1.8 ×10^5^ CPS. Interestingly, LS183_sig70c35lumplux_ showed relatively slower growth in LB media compared to the wild-type strain indicating that LumP expression had some metabolic constraint on the strain grown in the LB media (Figure 4D). This phenomenon was not shown by other SEn strains expressing the LumP. It reflected individual strain differences at the expense of the reporter expression. In the *in vitro* detection limit of the reporter expressing the LumP was improved by two-fold compared to the sig70c35 lux construct as determined by bioluminescent imaging of diluted cultures (Figure 5). The culture of OD_600nm_ 0.7 contained a density of SEn~2 ×10^8^ CFU/ml (a standard of all our SEn isolates).

### Characterization of putative virulence genes using the reporter sig70c35 lux (animal experiment 2)

**Figure 6:**
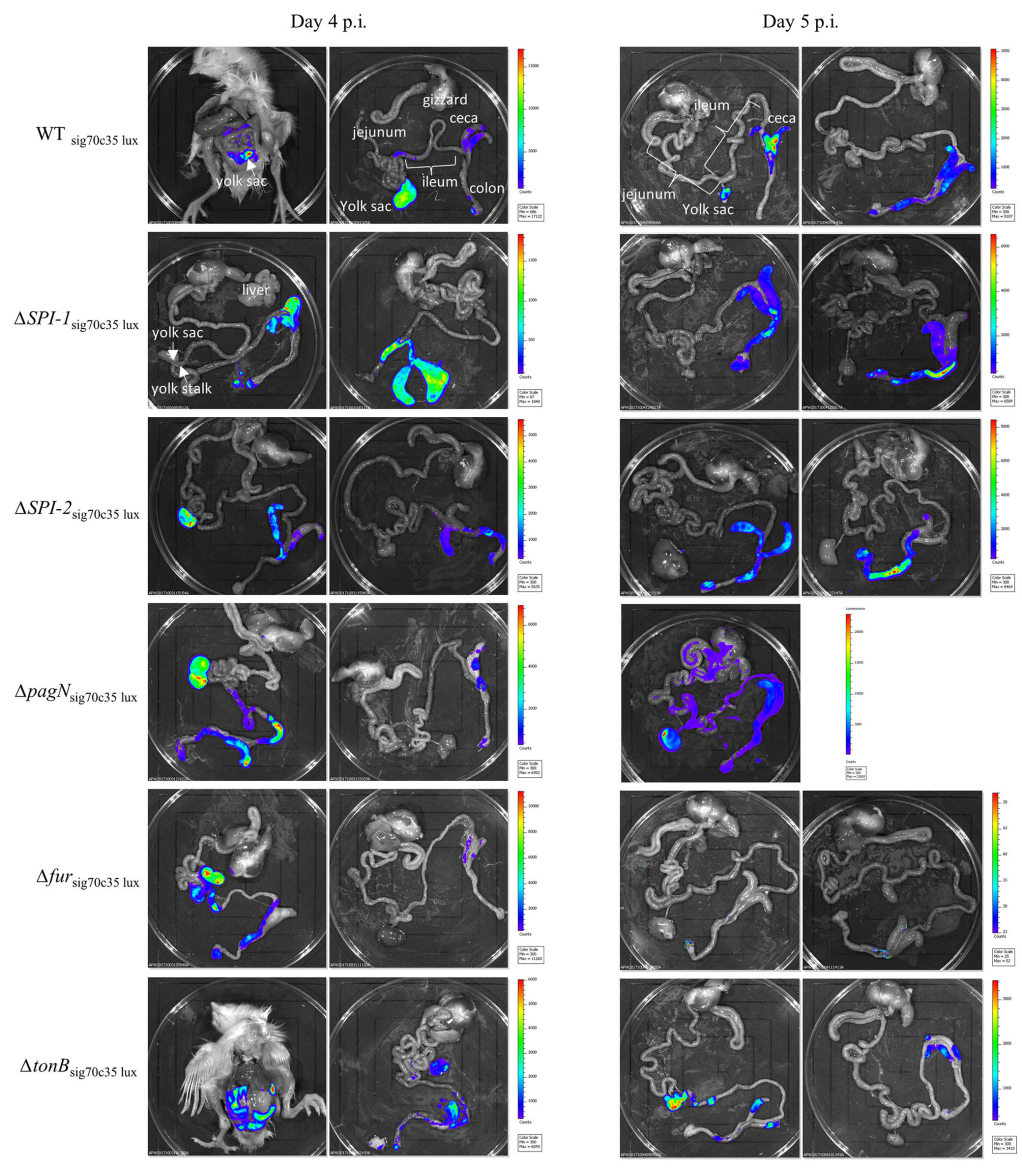
*Ex vivo* imaging of birds infected with wild type or mutants. The wild-type strain LS101 and isogenic mutant strains were tagged with the sig70c35 lux reporter. IVIS imaging setting: *f* = 1, exp = 30 s, binding = medium, and filter = open.

**Figure 7:**
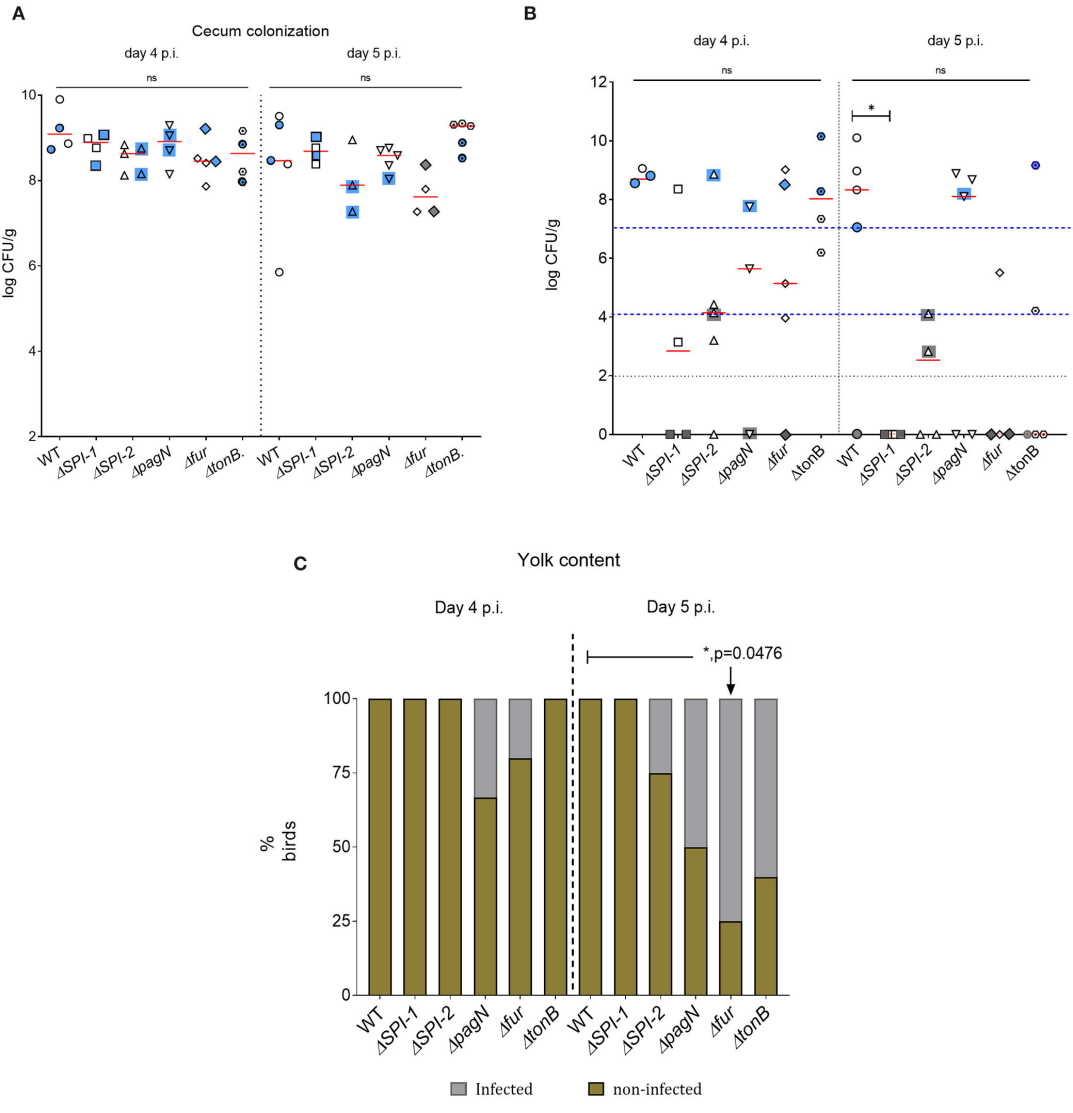
SEn burden by direct plating of cecal and yolk material. **(A)** cecum. **(B)** yolk. Each symbol represents a bird, and symbols with blue/gray color backgrounds are the birds selected for random imaging. Blue indicates that a bioluminescent signal was detected by imaging, while gray indicates no signals. The horizontal red bar is the median of the data set. Black dotted horizontal line indicates the detection limit of the brilliant green agar pate (100 CFU/g). Detection limit of the yolk sac by bioluminescent imaging was indicated by the blue dashed line. Mean colony count values of the cecum were statistically analyzed, while the yolk material median values were analyzed. The two blue lines in **(B)** stand for upper and lower margins of limit of detection by imaging, respectively. **(C)** Yolk sac infectivity after enrichments. ns, Not significant.

**Figure 8:**
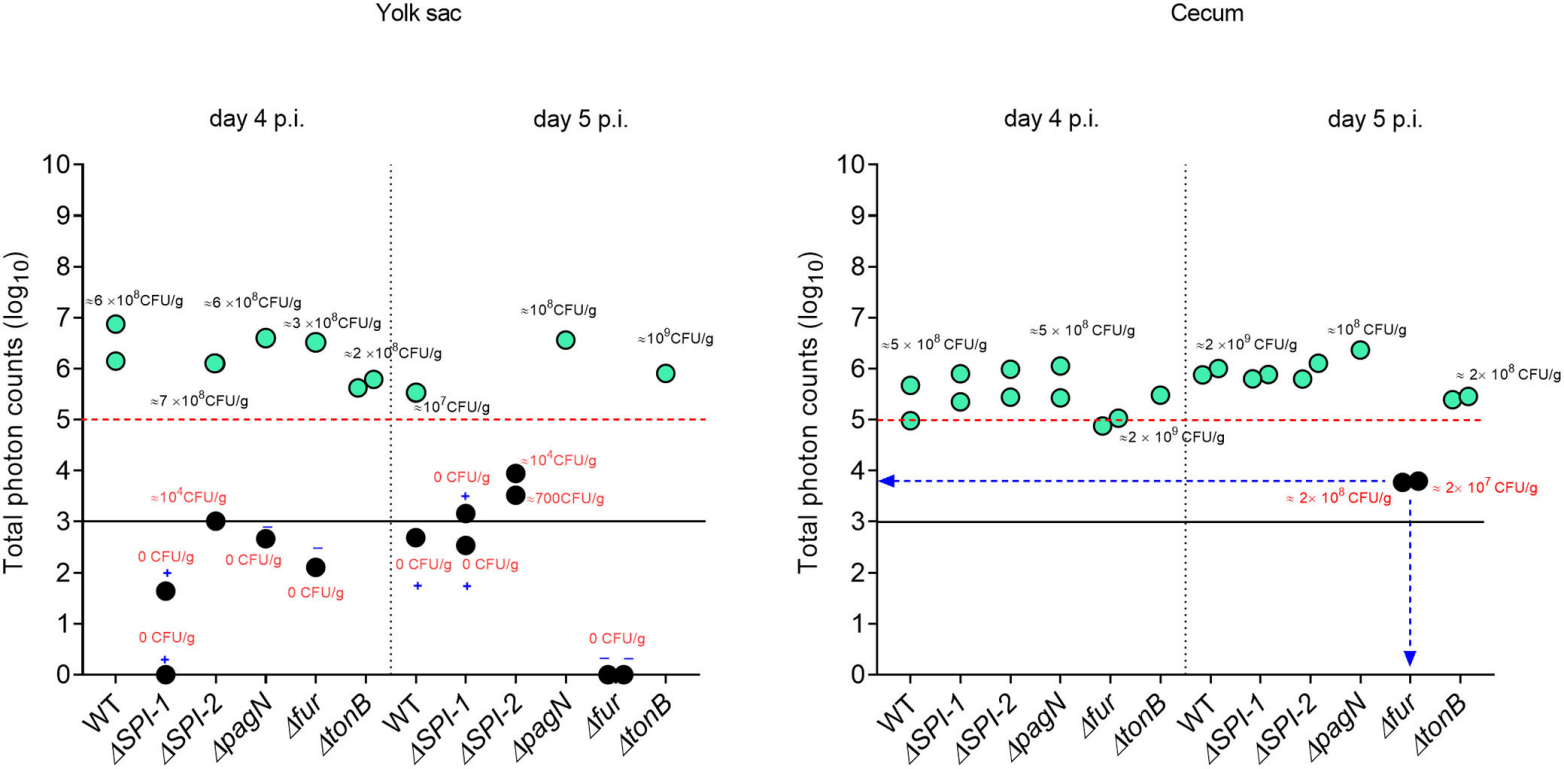
Relationship between total photons emitted from the organ and bacterial cell densities. Total photons are shown in log 10 scale. Each symbol represents a bird subjected to *ex vivo* imaging. Symbols highlighted in color represent positive for photonic detection while black symbols indicate no detectable photons. Some of the colony count values indicated proximity to the symbol. Infectivity of samples that underwent enrichment (samples with 0 CFU/g) is shown by + (infected) or – (non-infected). Black horizontal line is the background photon signal. Red horizontal bar represents minimum photon counts which are needed to for a bioluminescent signal to be detected from the surface of an image.

Five isogenic chromosomal mutants of SEnLS101 (chicken isolate), namely, Δ*SPI-1*, Δ*SPI-2*, Δ*pagN*, Δ*fur*, and Δ*tonB*, were tested in a chicken model to analyze the roles of these putative virulence genes in colonization of birds (Table 1). All the strains, including the wild type, carried the reporter sig70c35 lux to facilitate tracking by imaging. *Ex vivo* imaging of the gastrointestinal tract of the wild type infected bird showed (two birds per time point), bioluminescent imaging of cecum, ileum, and colon with surface intensities reaching to ~2,000 photon counts at day 4 p.i. (Figure 6). This surface intensity corresponded to a ~10^8^ CFU/g of SEn concentration in the cecum as determined by enumeration of cecal content (Figure 7A). Bioluminescent imaging was possible from all the birds examined from each group with intensities ranging from 1,000 to 2,000 photon counts on day 4 p.i. One exception to the observation was that surface intensities generated by one of the Δ*fur-*infected birds consisted of only 300 photons, which is relatively lower. However, the overall cecal SEn burden did not significantly differ in each group compared to the wild type; hence, signal intensity was generally comparable to the *Salmonella* densities on day 4 p.i. (Figure 7A). In addition to the gastrointestinal tract, a prominent bioluminescent signal was detected from the yolk sac of the wild type-infected birds. It consisted of signal intensities of ~5,000–~10,000 photon counts (Figure 6) with a density of~10^8^ CFU/g of an SEn burden (Figure 7B). Compared to the gastrointestinal tract, the yolk sac is a thin membranous structure that allows for more light emission from the surface. This may have been a reason for the high signal strengths from the yolk sac compared to the cecal tissue despite both having similar SEn densities. Overall, our bioluminescent imaging approach was able to show that in addition to the cecum, the yolk sac is a niche that SEn can colonize in young chickens after oral fecal infection. In comparison to the wild type-infected birds, two birds infected with Δ*SPI-1* did not show a bioluminescent signal from the yolk sac on day 4 p.i. (Figure 6). Direct plating of the yolk material from these birds did not result in any colony counts and hence were below the detection limit by imaging (Figure 7B). Compared to other mutants, Δ*SPI-1* showed a trend in low recovery from yolk material since the overall median level only reached ~10^3^ CFU/g on day 4 p.i (Figure 7B). The median SEn burden in the yolk for Δ*SPI-1*, Δ*pagN*, and Δfur day 4 p.i was ~10^4^ CFU/g, suggesting that these mutations also have decreased the virulence in causing yolk sac infection compared to the wild type (~10^8^ CFU/g). According to the available imaging data, detection limit in the yolk sac appeared to be between ~107 CFU/g and ~104 CFU/g (Figure 7B). We speculated that the detection limit of SEn infection in yolk sac by imaging might be less than that of SEn in the cecum, because it is more permissive for light penetration. We calculated the total photons emitted from the surface of the yolk sac and the cecum during bioluminescent imaging (Figure 8), which enabled us to estimate the minimum cell density required to facilitate bioluminescent imaging (Supplementary Table 1). The total photon count of ~10^4^ used as the background signal, as determined with organs, did not result in visually detectable bioluminescent hot spots (Figure 8). We hypothetically used the surface emission of 10^5^ photon count as the next available photon emission needed to generate a bioluminescent image. Based on this, minimum bacterial cell density was estimated, which was needed to generate an image. For the yolk sac infected with wild-type SEn, it was ~1 ×10^7^ CFU/g (1.1 + 07) day 4 p.i. and ~3 ×10^6^ CFU/g (3.48 + 0.07) on day 5 p.i. In the cecum, estimated cell density for detection by imaging was ~4 ×10^8^ CFU/g (3.99 + 0.08) on day 4 p.i. and ~1 ×10^8^ CFU/g (1.3 + 0.08) on day 5 p.i (Supplementary Table 1). So, overall, we predict that the detection limit of yok sac imaging will range from 10^6^ to 10^7^ CFU/g while being infected with the SEn strain LS101 with the sig70c35 lux reporter up to the age of 5-day-old. For the same age of birds, the detection limit in the cecum is ~10^8^ CFU/g.

**Table 1:** Putative virulence genes.

**Function**	**Gene region**	**Size**	**Function**
Salmonella Pathogenicity Island-1	SPI-1	40 kb	Invasion in to phagocytic and non-phagocytic cells. Encodes for a T3SS apparatus called T3SS-1 (16)
Salmonella Pathogenicity Island-2	SPI-2	40 kb	Intracellular survival in phagocytic cells. Encodes for a T3SS called T3SS-2
PhoPQ regulated gene N	*pagN*	720 bp	Hemagglutinin and invasin. Thought to contribute to invasion into mammalian epithelial cells. Function is not well characterized (17)
Ferric uptake regulator	*fur*	453 bp	Global iron regulator in Gram negative & positive bacteria. Acts as a repressor in iron excess conditions. De-represses the transcription of iron uptake systems at iron limiting environment (18)
Energy transducer TonB located in the inner membrane	*tonB*	729 bp	Part of TonB-ExbB-ExbD complex, transduces energy to receptors involved in the uptake of siderophores, vitamin B12, nickel complexes and carbohydrates (19)

Bioluminescent images of the cecum were obtained from all the groups on day 5 p.i. (Figure 6). There was no significant difference in cecal colonization between the wild-type and mutant strains on day 5 p.i. (Figure 7A). However, the *ex vivo* imaging of the cecum in the group infected with Δ*fur* did not generate a signal from the surface even if a concentration of 10^8^ CFU/g of SEn was reached (Figure 6). The Δ*fur* infected birds emitted ~ 6 ×10^3^ total photons, which was below the threshold for generating bioluminescent images. We speculate that in a Δ*fur* background, accumulation of intracellular sigma 70 factor might be low. Low translation of sigma70 factor will eventually lead to less RNA polymerase bound to the promoter region for transcription of the *lux* operon. This hypothesis needs to be investigated in future experiments. Detection of bioluminescence signals from the yolk sac was visible from the wild type-, Δ*pagN-*, and Δ*tonB-*infected groups on day 5 p.i. (Figure 6). However, except for Δ*pagN*, the median recovery numbers of SEn from the yolk content of Δ*SPI-1*, Δ*SPI-2* Δ*fur, and* Δ*tonB* was <10^4^ CFU/g, which is below detection by imaging (Figures 6, 7B). On most occasions, recovery of SEn from yolks in birds infected by mutants was below the detection limit by direct plating (Figure 7B). Enrichment data showed that yolk sac infectivity was significantly decreased by mutation in *fur* (25% infectivity) compared to the wild type (100% infectivity) on day 5 p.i. The mutation in *tonB* only showed 40% yolk sac infectivity, suggesting that Fe^3+^uptake systems may be involved in yolk sac infection.

### Kinetic of transmission can be traced in a seeder model using both reporters: sig70c35 lux and sig70c35 lump lux (animal experiment 3)

**Figure 9:**
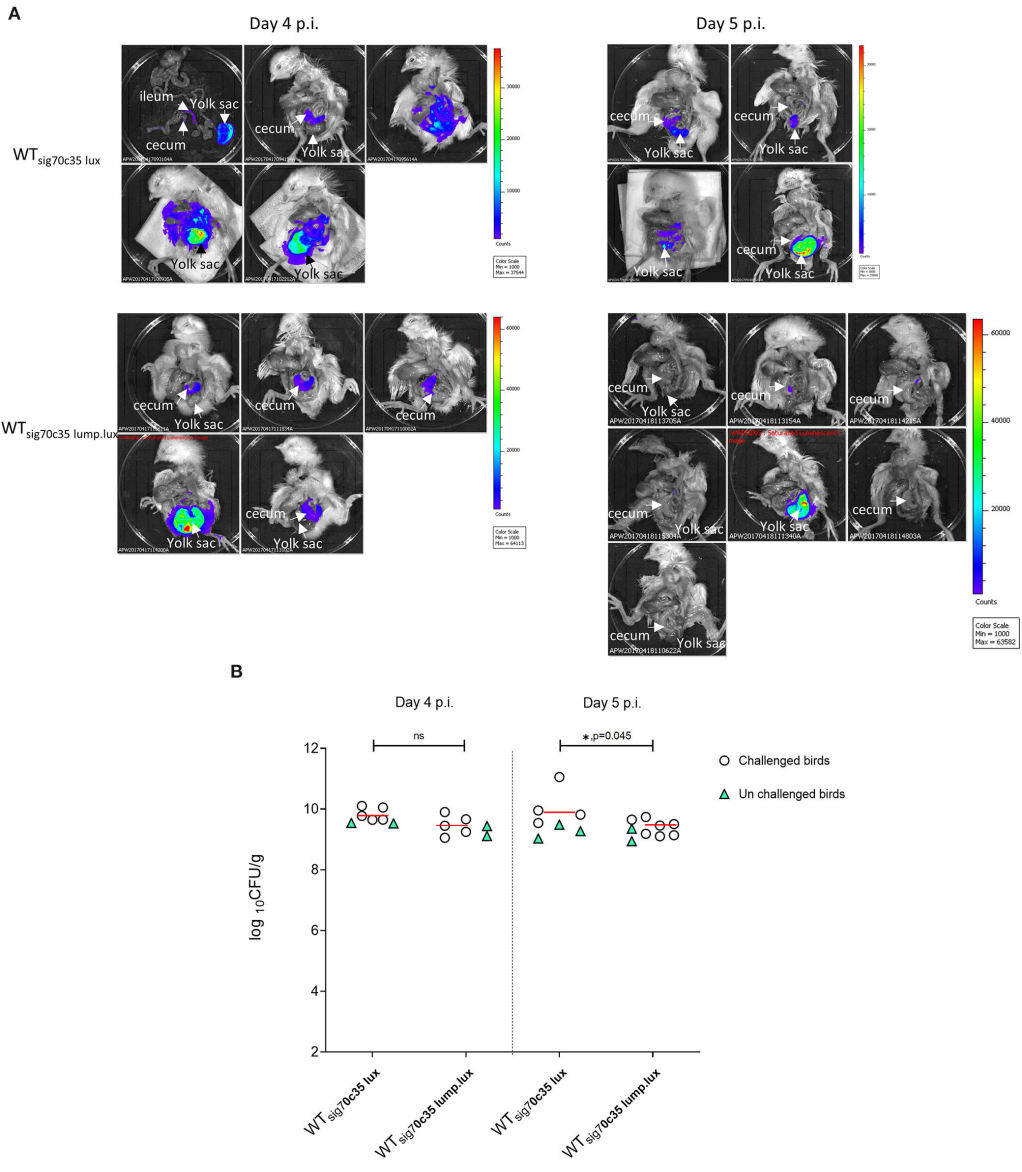
**(A)** *Ex vivo* imaging of the gastrointestinal tract by reporters. **(B)** Cecum bacterial cell densities. Each symbol represents a bird. The horizontal bar represents the median CFU/g value of challenged birds.

**Figure 10:**
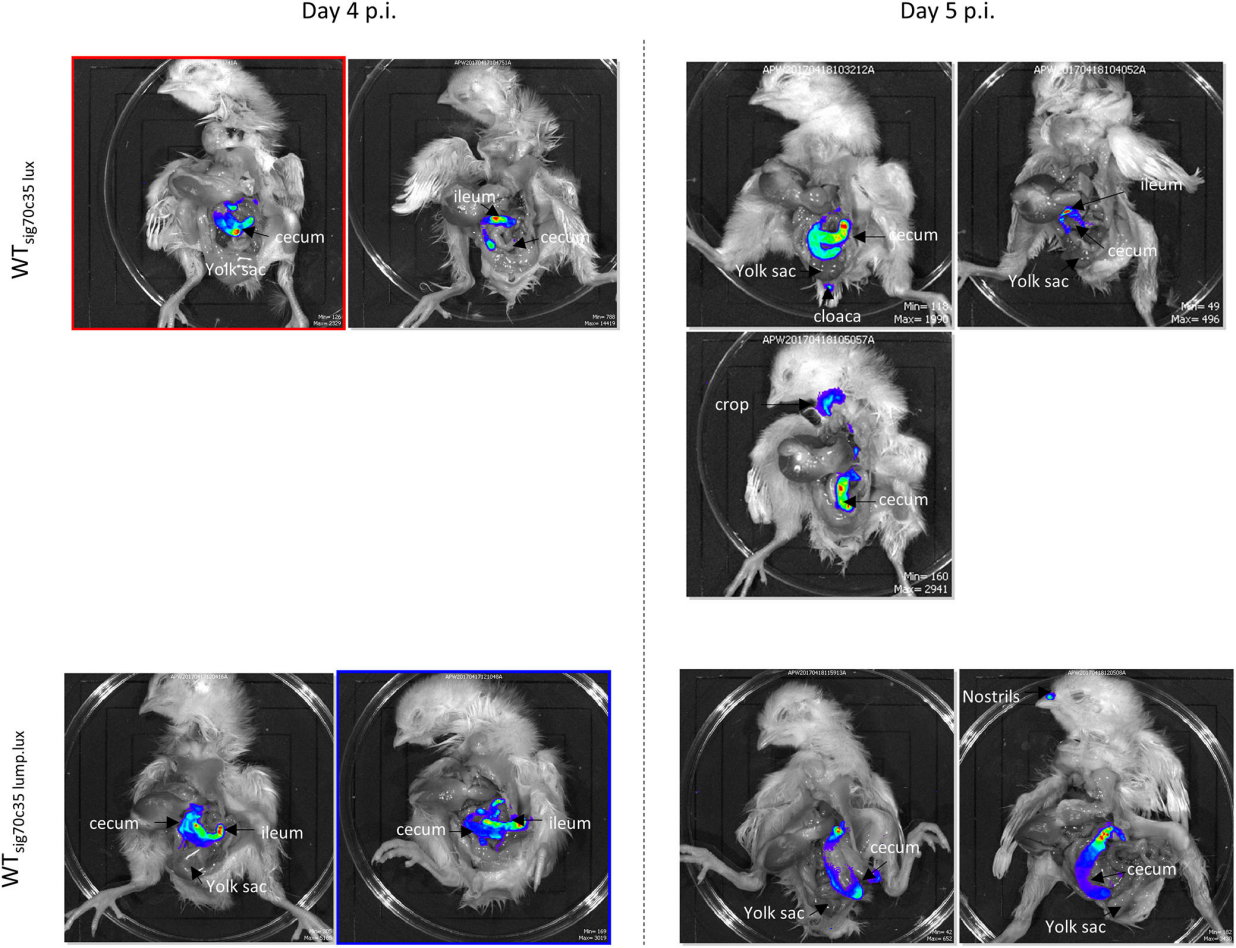
*Ex vivo* imaging of unchallenged birds infected by reporters. Signal intensities are displayed based on individual minimum and maximum photon counts.

**Figure 11:**
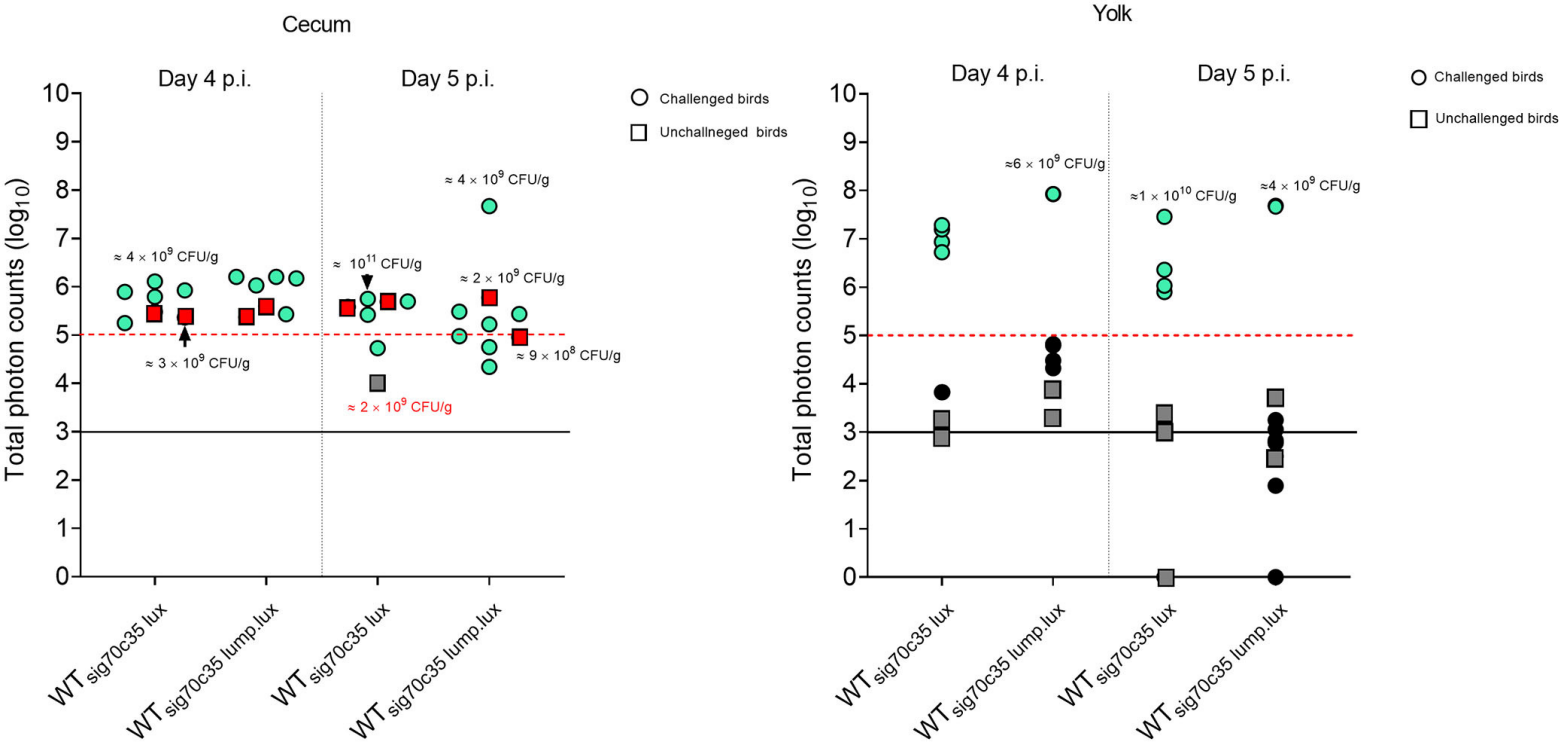
Relationship between total photons emitted from the organ and bacterial cell densities. Total photons are shown in log_10_ scale. Each symbol represents a bird that undergone *ex vivo* imaging. Some of the colony count values indicated proximity to the symbol. Red or blue color means positive for bioluminescent imaging, while dark gray to black is indicative of no images. Black horizontal line is background photon signals. Red horizontal bar is the minimum photons needed to be detected by bioluminescent imaging.

Two groups of day-old SPF birds were orally challenged (13 birds from each group) on the day of hatch; one group was infected with LS101_sig70c35lux_ (group A), and the other was infected with LS101_sig70c35lump.lux_ (group B). Each group had unchallenged birds: group A was left with five birds and group B had four birds. Sampling and whole animal imaging of the gastrointestinal tract were carried out on days 4 and 5 p.i. Consistent with our previous findings, the wild-type SEn carrying the sig70c35 lux reporter (WT_sig70c35lux_) emitted bioluminescent signals from the cecum and yolk sac regions of the challenged birds on day 4 p.i. In parallel, WT_sig70c35lux.lump_ with improved yield also facilitated imaging from the cecum and yolk sac. However, in contrast to the WT_sig70c35lux_, WT_sig70c35lux.lump_ only resulted in 1/5 birds with a visible yolk sac infection on day 4 p.i. (Figure 9A). So, most of the samples were below the detection limit of imaging of the yolk sac by WT_sig70c35lux.lump_. The measured SEn burden using the cecal material clearly indicated that the two reporter strains were able to colonize the cecum in compatible numbers (~10^9^ CFU/g) on day 4 p.i. (Figure 9B). Two imaged unchallenged birds on day 4 p.i. showed visually detectable bioluminescent signals from the cecum (Figure 10) and they were similar in strengths to the challenged birds (Figure 11), with the burden of *Salmonella* reaching ~10^9^ CFU/g of cecal material (Figure 9B). Bioluminescent signals of yolk sacs from the unchallenged birds on day 4 p.i. generated total surface photon counts of between ~10^4^ and ~10^3^, which were below the visual detection limit of 10^5^ total photons (Figure 11). The burden of SEn in the remaining unchallenged birds were maintained at ~10^9^ CFU/g on day 5 p.i. (Figure 9B) and produced bioluminescent images from the cecum (Figure 10). In parallel, yolk sacs from the unchallenged birds had total surface bioluminescent signals < ~10^4^ (Figure 11) and hence did not produce images with bioluminescent hot spots (Figure 10). The major way that the unchallenged birds had been infected with SEn was by ingestion of contaminated feed or water during this time. The crop material from one of the unchallenged birds on day 5 p.i. produced a bioluminescent image, which confirmed this mode of transmission (Figure 10). SEn-carrying reporters have been excreted into the litter, which was then picked up by the unchallenged birds at a certain time point. The level of colonization of the unchallenged birds was highly compatible to the level of the challenged birds in the two groups, indicating that both reporters had similar kinetics of transmission.

In contrast to the unchallenged birds, almost all the birds challenged with the WT_sig70c35lux_ reporter gave rise to detectable bioluminescent images with a surface emission of >10^5^ photons (Figure 11). Since most of the unchallenged birds had a total photon emission <10^4^ from the yolk sac with lack of detectable signals, it was highly unlikely that environmental transmission caused yolk sac infections in this study. Since the detection limit of yolk sac was estimated to be at ~10^6^ CFU/g (on day 5 p.i.) from our previous results, the yolk sac of the unchallenged birds may have had < ~10^6^ CFU/g in the seeder model. Toward day 5 p.i., two out of three birds had a total photon emission of <10^3^, which can lead to even 0 CFU/g by direct plating. Overall, our data suggested that the initial seeding of yolk sac through entry from the jejunum to the yolk *via* yolk stalk may have accounted for causing high frequencies of yolk sac infection rather than entry from the navel in the day-old chickens. The navel route of infection has been identified as the major route for yolk sac infection associated with bacteria. Hence, we conclude that the oral route provides a major entry point to cause yolk sac infection linked to SEn other than the navel.

We were able to calculate the minimum cell densities needed to obtain a bioluminescent image from the cecum, since we recorded the total photon emitted from the cecum and cell densities (Figure 11). On day 4 p.i., the detection limit of WT_sig70c35lux−_infected birds was ~10^9^ CFU/g (1.05 + 0.09), while for WT_sig70c35lump.lux_ it was ~3 ×10^8^ CFU/g (3.05 + 0.08; Supplementary Table 1). On day 5 p.i., the detection limit for the two reporters were as follows: ~10^10^ (9.6 5 + 0.09) and 4 ×10^7^ CFU/g (4.24 + 0.07; Supplementary Table 1). Our *ex vivo* imaging results indicated that WT_sig70c35lump.lux_ showed some improvements in detecting SEn *ex vivo* in the cecum relative to theWT_sig70c35lux_. The margin of detection of WT_sig70c35lump.lux_ in the cecum on day 5 p.i. extended to ~2 log improvement compared to WT_sig70c35lux_. However, the estimated limit of detection calculated for the WT_sig70c35lux_ strain in this experiment was higher than that what we have estimated from the earlier experiment (animal experiment 2), which was ~10^8^ CFU/g.

## Discussion

We hypothesized that colonization events of SEn can be detected by bioluminescent imaging (BLI) and that BLI can facilitate live bird imaging of day-old chickens. Our approach revealed that detection of bioluminescence signals was mainly possible from the gastrointestinal tract after performing a laparotomy. Detectable signals were localized in the cecum as early as 8 h p.i. and then again 30 h p.i. (Figure 3A). Furthermore, on days 4 and 5 p.i., the cecum was the major site for signal localization, confirming it is the predilection site for colonization of SEn (Figures 6, 9A, 11). Tracking photonic signal strength showed that the exact colonization event occurred beyond 24 h p.i. during infection with the sal18 strain (challenge dose of 10^9^ CFU). In an event of colonization, *Salmonella* will be replicative and metabolically active within the cecal environment (20). Also, the transcription rate of most essential genes including those located downstream of sigma 70-dependent promoters will be higher in a metabolically active status. Hence, the *lux* operon with a sigma70-dependent promoter should give rise to higher expression under these conditions. The dramatic increment of bioluminescent signal at 30 h p.i indicated that the population of SEn was relatively more metabolically active than at 8 h p.i. (Figure 3). Even though at 8 h p.i the load of *Salmonella* was comparable to that at 30 h p.i (10^8^ CFU/g), the elevated signal intensity suggested a more metabolically active status of bacterial cells at 30 h p.i. Our previous report showed that “active” colonization of the cecum with SEn strain LS101 occurred between 24 and 48 h p.i. in day-old birds challenged with the 10^9^ CFU dose (21). In that study, the cecal *Salmonella* burden reached a maximum of ~10^9^ CFU/g 24 and 48 h p.i. with surface photon ~5,000 counts imaged after 1 min of exposure. In this study, using the bioluminescent signal strength combined with colony counts, we were able to confirm our previous results that the most active SEn colonization occur >24 h <48 h p.i. Our approach also further stressed that BLI has the added advantage of defining key features of pathogenesis of *Salmonella* in chickens, such as temporal changes in colonization pattern.

The estimated detection limit in the cecum was ~10^6^ CFU/g using 1-day old SPF birds at 1 min exposure at 30 h of infection (Figure 3B). We saw that the estimated limit of detection was increased to the range of ~10^8^ CFU/g when image acquisition was set at 30 s of exposure on days 4 and 5 p.i. (Figure 8). In a series of experiments conducted by Rimet et al. (22), it was estimated that the limit of detection of *Salmonella s*trains (S. Typhimurium and *S*. Heidelberg) from the chicken cecum was >10^6^ CFU/g for reliable visualization. Hence, our estimated detection limits are in line with these findings. However, the limit of detection can vary significantly depending on reporter strength, experimental procedures, and exposure settings applied. For example, we have used a maximum of 1 min of exposure to avoid oversaturation as well as to minimize any low level of signals that are not reliable. The *lux* operon expressed under the sig70c35 promoter was strong enough to facilitate image acquisition at low exposure. In their study, Rimet et al. (22) used a long exposure time (3–5 min) for image acquisition optimized to the *lux* operon expressed under the EM7 promoter, which has been described by Burns-Guydish et al. (23). The EM7 promoter contains the sequences TTGACA in the “-35 region” (included in sig70c35) and TATAAT in the “-10 region” (not included in sig70c35), which are recognition consensus sequences for sigma factor family proteins. So, lux operon expression can be comparable between sig70c35 and EM7. However, the transposition of reporter into STm described in the previous study was located in the *hha* gene, which was disrupted during insertion, while in our study the reporter was placed in a permissive site, the *attTn7* site, downstream of the *glmS* gene. Hence it is unknown if the expression of the *lux* operon from two different sites in the chromosome has an impact on signal strength and *Salmonella* performance in the chicken milieu *in vivo*. Long exposure time will be necessary as described by Rimet et al. (22) to image older birds (42 days old) as the tissues become less transparent. Longer exposure time using the reporter sig70c35 lux has not been conducted in this study but will be an option to improve the sensitivity. In this study, we have maintained the anaerobic environment of the cecum while capturing photon detection. To our knowledge, similar procedures have not been documented in other studies and an evaluation of the whole gastrointestinal tract has not been published so far. Since oxygen is needed to generate a light signal, BLI of intact organs or aerated tissues may result in different detection limits.

The total of 10^6^ CFU or the concentration of ~10^8^ CFU/g of STm in the cecum is considered as the limit of detection during *in vivo* imaging of mice (5). These results are from the first report of monitoring infection by BLI of a living animal tissue (5). In contrast to a chicken infection model, live BLI was possible using mice in which signals were mainly localized to the cecum throughout the study period (8 days) (5). The authors used the STm strain SL1344 carrying the plasmid-encoded *lux* operon of *P. luminescence*, which was constitutively expressed from the *lac* promoter. The Lac promoter consensus contains TTTACA in the−35 region and TATGTT in the−10 region. The strength of the signal generated under these conditions prohibited visualization of extraintestinal infection (liver, spleen and lung) since the bacterial load was below the detection limit (<10^6^ CFU/organ). Shivak et al. (11) were able to show that STm carrying the *lux* expression under sig70c35 facilitated *ex vivo* imaging from the liver and splenic compartment; however, the detection limit has not been documented (11). In contrast to imaging of mice, chickens infected with the SEn strain LS101_sig70c35_ lux did not result in any BLI from surfaces of the liver or spleen, and most of the birds carried <10^5^ CFU/g of *Salmonella* (Supplementary Figure 2A). The relatively large size of organs compared to mice and absorption of the blue light by blood may require higher bacterial numbers to be detected in the liver and spleen of chickens.

For the first time, we report the BLI-facilitated detection of yolk sac infection with SEn after oral gavage (with a detection limit of >10^6^ CFU/g using the sig70c35 lux construct; Figures 6, 9). The yolk sac consists of a yolk material covered by a vascularized thin membrane structure called yolk sac membrane. So, it is more transmembrane than the gastrointestinal tract. In fact, the estimated detection limit during yolk sac infection by LS101_sig70c35lux_ was much lower than in the cecum (~1–2 log difference in CFU/g). The yolk sac membrane resembles the intestinal epithelium with microfold for nutrient transport during embryonic stages. The absorptive function rapidly decreases after hatch, but the residual yolk material gets transported through the yolk stalk to the jejunoileal junction (24). This short channel will also provide the opportunity for bacteria to enter the yolk from the jejunum. SEn reached detectable levels to be visualized by BLI on day 4 p.i in most of the orally gavaged birds (Figures 6, 9A) and was not detected in any of the unchallenged birds examined (Figure 11). In parallel, an experiment from our previous study provided evidence that yolk sac infection was not visualized prior to day 2 p.i. by LS101_sig70c35_ [Supplementary Figure 4 of (21)]. The passage of the yolk stalk gets partially occluded by lymphocyte proliferation minimizing the transfer of material between the yolk and the jejunum (25). Altogether, it is highly likely that SEn had translocated from the intestine (at the jejunoileal junction) to the yolk material between days 1 and 3 p.i. and replicated to >10^6^ CFU/g by day 4 p.i. to be visualized.

Incidences of yolk sac infection with different *Salmonella* serovars has been reported and led to yolk sac retention in commercial broilers (26). Recently, it has been shown that oral inculcation of *E. coli* (9.5 ×10^2^ CFU/bird) was able to cause yolk sac infection with a mean value of 144 CFU/g of on day 2 p.i and persisted up to day 14 (mean value 10^4^ CFU/g) (27). Oral inoculation of *Salmonella* Heidelberg (SHb) and SEn (phage type 4) in day-old SPF chicks resulted in lesions due to yolk sac infection on days 7 (SHb and SEn) and 14 p.i (SHb) (28). However, quantification of the *Salmonella* burden in the yolk sac has not been reported in the study (28). In the same study, the SEn strains did not appear to cause yolk sac-associated lesions as early as day 2 p.i., but the experiment was not designed to asses any presence of *Salmonella* (28). Our data support that the yolk sac can act as a colonization niche for *Salmonella* following oral infection, which can replicate to a level of detection by BLI on day 4 p.i. Future studies are warranted whether oral challenge of SEn can cause clinical manifestations and lesions linked to yolk sac infection. Intra-navel inoculation of avian pathogenic *E. coli* (APEC) strains has been the popular method for developing yolk sac infection-associated mortality and lesions to test therapeutics (29, 30). The model has many merits such as requirement of low challenge dose and reproducibility of clinical signs, lesions associated with yolk sac infections. In fact, an unhealed navel is considered a major route of infection along with defects in hatchery practices. However, we caution that in a healthy flock population, the oral route of infection will play a major role in disease pathogenesis and birds will be susceptible to yolk sac infection between days 1 and 4 post hatch. Virulence determinants of *Salmonella* serovars in causing yolk sac infection have not been thoroughly investigated and research on this avenue will facilitate the discovery of potential therapeutics against the ever-growing number of *Salmonella* serovars but should not be limited to *Salmonella*.

Mutant strains carrying the reporter (sig70c35 lux) facilitated the characterization of SEn within the gastrointestinal tract as well as in extraintestinal tissues such as yolk sac. Imaging data (Figure 6) and the bacterial quantification of the cecal material (Figure 7A) confirmed that cecum colonization was not affected by deletion of each gene alone. However, birds that were imaged on day 5 p.i. infected with Δ*fur* were not able to produce detectable photons, indicating a colonization defect that did not correlate with the actual colony count (Figure 8). It is plausible that in a Δ*fur* background, transcription of most of essential genes may not be regulated by sigma 70 factors. Deletion of ferric uptake regulators will directly interfere with normal iron hemostasis in the bacterium and further inhibit the activation of some detoxification mechanisms against reactive oxygen and nitrogen species. Therefore, we hypothesize that in a *fur* mutant background SEn may undergo a stressed condition in coping with the cecal environment, which in turn activates alternative sigma factors to drive metabolism and survival.

In contrast to the cecum, signal localization in yolk sacs infected with isogenic mutants showed a positive correlation with bacterial load. The lack of photonic detection from the yolk sacs of birds infected with Δ*SPI-1* on days 4 and 5 p.i (Figure 6) was in agreement with the fact that we did not harbor SEn by direct plating (Figures 7B, 8). There was a significant difference in cecal colonization level on day 5 p.i, as none of the yolk sacs had colony counts by direct plating (Figure 7B). SPI-1 is a 40-kb size locus encoding mainly genes related to the type 3 secretion system-1 (T3SS-1) apparatus and effectors secreted through T3SS-1. It is well known that effectors secreted through T3SS-1 trigger actin polymerization of the host cell and facilitate invasion into vertebrate cells. The locus also bears a metal uptake system encoded by *sitABCD*. This is the first report to show that the SPI-1 of a *Salmonella* serovar contribute*s* to yolk sac colonization after oral infection in chickens. It has been previously hypothesized that high SPI-1 expression during growth of SEn in egg yolk can potentiate virulence (31). Experimental evidence whether egg yolk conditions enhance induction of SPI-1 is currently unknown. The exact mechanism of how the SPI-1 locus contributes to yolk sac infection and colonization remains to be investigated. We suggest that SPI-1 might aid in effective colonization and replication in the jejunum, which induces more bacteria to swim toward the egg yolk. However, all the birds that were below the detection limit by direct plating remained 100% positive after yolk enrichments on day 5 p.i. (Figure 7C).

Furthermore, the enrichment data showed that Δ*fur* led to significant lower infectivity of yolk sac on day 5 p.i compared to the wild-type strain (Figure 7C). In parallel, the BLI of Δ*fur*-challenged birds did not show detectable photonic signals from the yolk sacs on day 5 p.i. (Figure 6). Deletion of the global iron regulator will enhance iron uptake because fur acts as a repressor for iron uptake systems. However, retractable iron influx will generate reactive oxygen species through the Fenton reaction that is toxic to bacterial cells. The number of superoxide dismutation enzymes is regulated by Fur (18), hence Δ*fur* may be more susceptible to killing by reactive oxygen species (ROS). It is currently unknown which mechanisms may be behind this phenomenon and how Fur is involved in yolk sac infection and colonization. Our data suggested that deletion of *fur* significantly affects colonization in the yolk sac, which is in contrast to the situation in the cecum because the bacterial burden in the cecum was not affected. Yolk sac infection with a SEn strain devoid of functional TonB-dependent systems also showed a trend in reduction of yolk sac infection on day 5 p.i (Figure 7C). A mutation of *tonB* completely blocks internalization of Fe^3+^ siderophores from the environment. Almost all ferric iron can be bound to a ligand or proteins in the host. It is known that yolk contains ovotransferrin and lactoferrin, which can strongly bind ferric iron; hence, secretion of high affinity Fe^3+^ chelators such as enterobactin and salmochelin will be advantageous for growing efficiently in yolk. Summarily, ferric uptake will be involved in colonization of the intestinal milieu that facilitates migration of *Salmonella* from the jejunoileal junction to yolk. Since mutations of *tonB* have a pleotropic effect not limited to ferric uptake, future experiments are warranted using specific mutations in siderophore secretion (Δ*entB* and Δ*iroB*) to delineate the role of enterobactin and salmochelin in yolk sac infection by SEn. However, we suggest that recent advancements in vaccination strategy to induce high titers of anti-siderophore antibodies in eggs of layers (IgY) will constitute a promising avenue to protect chickens against yolk sac infection by SEn (32).

The blue shift in the “Lump. Lux” construct described in this study (Figure 4C) is mediated by excitation of a noncovalently bound fluorophore, 6,7-dimethyl-8-ribityllumazine (DMRL/lumazine) in the LumP, which interacts closely with luciferase and luciferin; however, a mechanistic view on obtaining a relatively high quantum yield is unknown (15, 33). Genes encoding the LumP are naturally present in *Photobacterium* species in opposite direction to the *lux* operon under the same promoter. In these species, the *rib* operon (riboflavin synthesis) is transcribed with the *lux* operon to ensure the supply of FMN (riboflavin-5'-phosphate) and DMRL molecules to the light-emitting reaction (34). In contrast, *Photorhabdus* lacks a coding sequence for the LumP, and the *rib* operon is not co-transcribed with the *lux* operon. Here, we showed that co-transcription of *lump* from *Photobacterium leiognathi* and the *lux* operon from *Photorhabdus luminescens* not only shifted the wavelength but also increased the signal intensity (Figure 4). This was possible because luciferases from various luminous bacteria have high sequence homology between each other (35). The *in vitro* detection limit of SEn carrying the sig70c35 lump.lux was improved by ~1 dilution (two-fold) compared to the original construct (sig70c35 lux; Figure 5) and followed a similar trend *in vivo* by improving the limit of detection to ~10^7^ CFU/g (Supplementary Table 1). Future experiments are needed to accurately determine the detection limit. We suggest the improved reporter module (lump.lux) has more implication in gene regulation/ expression studies than expressing them constitutively. We assume that the constitutive expression of both lump and lux may have a fitness cost in replication of *Salmonella* in extraintestinal niches. For example, most of the birds infected with LS101_sig70c35lump.lux_ did not show a detectable level of photons from the yolk sac compared to those infected with LS101_sig70c35lux_ on days 4 and 5 p.i. (Figure 11). The median value of SEn concentration in the liver and spleen infected with LS101_sig70c35lump.lux_ was <10^4^ CFU/g of tissues, which was lower than the concentration of LS101_sig70c35lux_ in the infected birds (>10^4^ CFU/g; Supplementary Figure 2B). In contrast, cecal colonization level among infected groups by each reporter was comparable (Figure 11). In the future, co-infection experiments are needed to analyze any fitness cost associated with the need to constitutively express bioluminescent reporters in day-old chickens.

In summary, our effort to track SEn infection with the help of BLI facilitated the understanding of a colonization event and allowed for localization of signals in the gastrointestinal tract. This was enabled by *ex vivo* imaging of the excised gastrointestinal tract. Detection of bioluminescent signals emitted from the gastrointestinal tract from intact birds was not possible. This is largely because of anatomical barriers that absorb or block the light traversing to the surface. Bacterial bioluminescence has the potential to be improved by transfer of energy to a linked fluorescent molecule. As a proof of concept, co-transcription of the *lux* operon with *lump* boosted the signal strength without any need of a linker. The improved light-emitting module called sig70c35 lump.lux can be used in gene expression analysis in the future because of improved detection as determined by *in vitro* culture. For the first time, yolk sac infection was visualized by BLI during SEn infection after oral uptake. The anatomical location of the yolk sac may favor visualization of bioluminescent bacteria by live imaging during yolk sac infection. In an event of clinical manifestation, an enlarged yolk sac can move toward a more ventral-dorsal position side close to the skin, alleviating the blockage from the intestines and pectoral muscle. This, in turn, will facilitate photonic detection from the ventral side of the birds. Among putative virulence determinants, mutations in *SPI-1, fur*, and *tonB* showed clear trends in decrease in colonization, which were correlated with the lack of detectable photons. However, the exact mechanisms on how each of these factors contribute to yolk sac infection remain to be fully elucidated.

## Materials and methods

### Bacterial strains, media, and growth conditions

*Salmonella enterica* ssp. *enterica* serovar Enteritidis strains Sal18, LS101, and LS183 were used in this study. All the strains are poultry isolates recovered during *Salmonella* outbreaks in Canada. Regarding Sal18, numerous studies have been performed and published from our laboratory. The virulence potential of LS101 and LS183 was tested in a separate co-infection trial using 3-week-old birds (unpublished data). We found that both LS101 and LS183 showed higher potential for systemic infection and persistence than Sal18, measured as increased prevalence in the liver and spleen of orally challenged chickens. Some of the cloning experiments were performed on *E. coli* DH10B (pCS26-Cm^r^) or *E. coli* CC118 (pUC18R6K-mini-Tn7T-Cb^r^) provided by Aaron P White VIDO from University of Saskatchewan. Plasmid purification was performed using a Qiagen MiniPrep kit according to the manufacturer's guidelines. All the cloning strains were inoculated from frozen stocks into a Luria Bertani (LB) agar supplemented with appropriate antibiotics (Cm, chloramphenicol 9 or 34 μg/ml; Cb, carbenicillin, 50 μg/ml). A complete list of the bacterial strains and plasmid used in this study can be found in Supplementary Table 2.

Luciferase expression assays were conducted as described previously using a Victor V^3^ multilabel plate reader (Perkin-Elmer) (11). Here, the expression of the *lux* operon was measured by means of intensity of light signal. Briefly, overnight cultures of *E. coli* or *S*. Enteritidis strains carrying *luxCDABE* expressed under a constitutive sigma promoter were diluted 1:600 in an LB broth to a final volume of 150 μl (with or without antibiotics) on 96-well clear-bottom black plates (9520 Costar; Corning Inc.). The culture in each well was overlaid with 50 μl of mineral oil prior to the luciferase assays. The plate reader measured the absorbance (580 nm, 0.1s), luminescence (1s, counts per second CPSs) every 30 min during growth at 37°C with agitation.

*Salmonella* Enteritidis challenges were prepared from a frozen stock propagated on LB plates with an appropriate antibiotic (Cm 34 μg/ml). Single colonies were grown overnight in 5 ml LB medium at 37°C with agitation. Overnight cultures were then subcultured in LB at 37°C with agitation to gain an OD of 0.7 (2 ×10^8^ CFU/ml). Cells were harvested by rotation at 7,000 rpm and 37°C and resuspended in 1XPBS (Gibco, PH-7.2).

### Chromosomal transposition of the reporter -sig70c35 *luxCDABE*

The method is based on the mintn7T transposon system described by Shivak et al. (11). Briefly, pHSG415 (12kb) and temperature-sensitive plasmid (helper vector) encoding transposon genes (*tns-ABCD*) were electroporated into SEn strains. Successful transformants were grown from LB plates supplemented with carbenicillin (50 μg/ml) at 30°C. Electro competent cells harboring pHSG415-*tnsABCD* were prepared to be used for electroporation with pUC18R6K-mini-Tn7T (delivery vector contained the sig70c35 *luxCDABE*). Following recovery in the SOC medium, bacterial cells were agitated at 30°C for another 1.5 h to facilitate integration of the *lux* operon at high frequency into the *attTn7* site of bacteria by transposition. Then, the cells were recovered on Cm (9 μg/ml) LB agar plates at 37° overnight to remove the temperature-sensitive pHSG415. Colonies were imaged with IVIS Lumina II for light production and further confirmed by PCR using primers described previously (11). A complete list of the primers used in this study is shown in Supplementary Table 3.

### Generation of delivery vector containing *lump* upstream of *lux* operon for expression

A genetic fragment containing *lump* (*P. leiognathi*) was amplified using the Taq polymerase from the plasmid pSKB3-GBD-Lump (36) purchased from addgene (cat: 65894), and cloned into the pGEM T vector (Promega). A forward primer was designed to contain a *Bgl*II overhang plus a Shine-Dalgarno sequence for ribosome binding (Supplementary Table 3). The reverse primer included two stop codons and was flanked by *Bam*HI (Supplementary Table 3). Both the *Bam*HI and *Bgl*II enzymes recognize similar palindromic sequences (GGATCT and AGATCT respectively) and digestion will result in exact same overhangs. The amplicon was digested with *Bam*HI and *Bgl*II to ligate into pCS26-Cm^r^sig70c35 *lux*(DH10B *E coli*), which was linearized by digestion of *Bam*HI. Successful ligation of *lump* was screened by colony PCR using the pZE05 and pZE06 primers (Supplementary Table 3). The ligation resulted in two orientations of *lump*. Only forward orientation compatible with transcription by the sig70c35 promoter was selected by performing enzyme digestion using BamHI and XhoI (band patterns 9,567 and 776 bp). Plasmids were also sequenced from the pZE05 and pZE06 primers (Sanger sequencing at Plant Biotechnology Institute, University of Saskatchewan). The resulting pCS26-Cm^r^ sig70c35 lump.lux plasmid was digested with the PacI enzyme and the reporter construct (~8.7 kb; Cm^r^ sig70c35 lump.lux) was ligated into the *Pac*I-digested pUC18R6K-mini-Tn7T (empty vector, modified to have a PacI restriction site; Cb^r^). Potential clones were selected by growth on LB agar supplemented with 50 μg/ml Cb and 9 μg/ml Cm. Ligation into the mini-Tn7T vector generated two orientations of the reporter, but reverse orientation was selected (direction of the transcription occurs Tn7 left to Tn7 right). To confirm that the constructs were ligated in the desired orientation, plasmids were digested with NotI -HF, XbaI, and EcoRV-HF, visualized, and resulted in a band pattern from agarose gel electrophoresis. The most prominent bands of ~4, ~3.8, and ~1.6 kb visualized in 1% agarose gel confirmed the reverse orientation.

### Generation of gene knockouts

The Lambda Red system, which was described by Datsenko et al. (37), was utilized to create gene knockouts and replacement by an antibiotic marker (refer to Table 1 for the description of selected genes). Briefly, strain LS101 was transformed with temperature-sensitive pKD46, which encoded red recombinase enzymes ⋎, β, and *exo* induced under L-arabinose. Fresh electro-competent cells containing pKD46 (50 μl of aliquots, induced by L-arabinose) were made to be used for subsequent transformation of PCR products for gene replacement. Primers were designed based on the complete genome sequence of a PT4 *Salmonella enterica* subsp. *enterica* serovar Enteritidis strain deposited in NCBI (NC_011294) (38). The PCR products generated by gene replacement had the Cm marker from the pKD3 flanked by a flippase recognition sequence and a 49-nucleotide homology extension of the gene of interest. Gel-purified PCR products (100–500 ng) were electroporated into LS101.pKD46 cells and cells recovered in an SOC medium at 37/30°C for 2–3 h and plated in 9 μg/ml of Cm. Cells of colonies containing the anticipated mutations were subjected to PCR, and PCR products were sequenced for further verification. The Cm marker was cured with pCP20 after transformation with the pCP20 plasmid (39, 40).

### Chicken infection model

SPF eggs were purchased from Charles River Laboratories and incubated until the hatched at the VIDO animal facility. All the birds were raised at the VIDO-animal care facility in direct accordance with guidelines drafted by the University of Saskatchewan's Animal Care Committee (UACC) under Canadian Council on Animal Care (CCAC). On the day of hatch, swabs were collected to check for any *Salmonella* contamination before transferring the animals to separate rooms. The birds were not caged and had access to litter. *Ad libitum* feed and water were provided.

#### Animal trial 1

One-day old SPF birds were raised in three different rings in a room to accommodate three different concentration of challenge doses assigned to each group. Each group had 5 healthy birds before the challenge. Three doses of SEn strain Sal18 was prepared: ~2 ×10^5^, ~2 ×10^7^, and ~2 ×10^9^ CFU/ml. One-day old birds were orally gavaged with 0.5 ml of the assigned dose of Sal18. One bird from each group was euthanized at 2, 4, 8, and 24 h p.i., and BLI was performed. Samples from the liver (part of the liver) and spleen (whole organ) and cecal contents were weighed and homogenized (only liver and spleen) in saline (0.85% sodium chloride), and serial dilutions were plated onto brilliant green agar plates (BGA) to determine bacterial counts. In addition, aliquots from homogenates from the liver and spleen were enriched by incubation in a selenite broth overnight at 37°C. The enriched samples were streaked onto BGA to single colonies to determine whether they contained each *Salmonella* strain inoculated.

#### Animal trial 2

After hatch, eight to nine birds were assigned to six different rooms before the oral challenge with generated five mutant strains of LS101 carrying the reporter sig70c35 lux. One-day-old SPF birds from each room was orally challenged with 10^9^ CFU from one mutant strain, which was assigned to the room. One group was challenged with the wild-type strain carrying the reporter sig70c35lux. Birds were euthanized for BLI and sampling. On days 4 and 5 p.i., BLI was performed before tissue samples were collected for bacterial enumeration. Two birds/room were randomly picked for BLI on each day. Liver, spleen, and cecal materials were collected as stated in the animal trial for bacterial enumeration on days 4 and 5 p.i. Apart from that, any other tissue with prominent signals was collected for enumeration. In our study, the yolk material was collected for bacterial quantification by direct plating.

#### Animal trial 3

One-day old SPF birds were assigned into two groups and raised in two separate rooms. One group of birds was assigned to be infected with the LS101_sig70c35lux_ strain; nine of the 16 birds in this group was orally gavaged (10^9^ CFU/bird) with the LS101_sig70c35lux_ strain, and the remaining seven birds remained unchallenged and comingled with other infected birds. The second group of birds was assigned to be challenged with the LS101_sig70c35lump.lux_ strain; 12 of the 16 birds from this group was orally gavaged (10^9^ CFU/bird) with the LS101_sig70c35lump.lux_ strain, and the remaining four remained unchallenged. BLI and sampling were performed on days and 5 p.i. The BLI and sampling procedure was same as in animal trial two except for some differences. In this experiment, all the birds were subjected to BLI at the end of the trial. Only selected yolk materials were enumerated for bacterial quantification.

### *Ex vivo* imaging with whole animal imager

First, a whole bird was imaged after being positioned to capture any surface signal emitted from the gastrointestinal tract through the skin. After that, laparotomy was performed to excise the gastrointestinal tract to be laid out on a large Petri dish to visualize the signal emitted from the surface of the organ. After initializing the instrument (IVIS Lumina II by Caliper Life science), the bioluminescent setting was selected (Filter-open). To maximize the capture of low bioluminescent signals, the size of the aperture was set to 1 (*f* = 1). In the first experiment, photons were detected by auto exposure settings (maximum 60 s). The next couple of animal exposures was set at 30 s with medium binning to create the images. The total photon count emitted from each cecal compartment or yolk sac was measured using the region of interest tool (ROI) provided by the Living imaging software (version 4.0). The region of interest was selected by drawing exact boundaries around the organ using the free drawing tool.

### Data analysis

A statistical data analysis was conducted using the GraphPad Prism software version 8.4.2 under the institutional license provided to Vaccine and Infectious Disease Organization. Non-parametric tests were conducted to select an appropriate test method because the data were not normally distributed. Mean comparison of the colony counts between the wild type and the mutant was performed by the Kruskal–Wallis test. The Mann–Whitney was conducted to compare the median difference when mentioned. The Fisher's exact test was conducted to analyze enrichment data using fractions of infected and noninfected birds. The statistical significance of all the methods was set at *p* = 0.05.

## Data availability statement

The datasets presented in this study can be found in online repositories. The names of the repository/repositories and accession number(s) can be found in the article/Supplementary material.

## Ethics statement

The animal study was reviewed and approved by University of Saskatchewan's Animal Care Committee (UACC).

## Author contributions

DW and WK designed the study and wrote the manuscript. DW, P-KL, and WK collected the experimental data and analyzed the data. BA and AW discussed the data and edited the manuscript. All authors contributed to the article and approved the submitted version.

## Funding

This work was supported by Alberta Agriculture and Forestry (2018F145R), Chicken Farmers of Saskatchewan (KOE2018002), Egg Farmers of Alberta (2018F145R), Canadian Poultry Research Council (KOE2018002), and Chicken Farmers of Canada (1433-19). Financial support to DW was provided by the Devolved scholarship granted by the department of Veterinary Microbiology- WCVM.

## Conflict of interest

The authors declare that the research was conducted in the absence of any commercial or financial relationships that could be construed as a potential conflict of interest.

## Publisher's note

All claims expressed in this article are solely those of the authors and do not necessarily represent those of their affiliated organizations, or those of the publisher, the editors and the reviewers. Any product that may be evaluated in this article, or claim that may be made by its manufacturer, is not guaranteed or endorsed by the publisher.
