# Supplementary material for: Characterization of colonization kinetics and virulence potential of Salmonella Enteritidis in chickens by photonic detection
Source: Front Vet Sci. 2022 Aug 2;9:948448. doi: 10.3389/fvets.2022.948448 (PMC9378992; doi:10.3389/fvets.2022.948448)
Supplement: Supplementary file 1 [file Data_Sheet_1.docx]

**SUPPLEMENTARY FIGURES AND TABLES**

**Supplementary figure 1.** Growth in a plate reader. Overnight cultures of wildtype (WT) or wildtype tagged with sig70c35 lux reporter grown in 1:600 dilution in a 96 well plate to measure optical density (OD). Three technical replicates from each strain were used at a time. Each line indicates a replicate. Assay was performed 4 times. After 10 hours, temperature switched to 42 ^0^C. *Salmonella* Enteritidis isolate Sal18 was used.

**Supplementary figure 2. SEn burden in liver and spleen. A)** After infection of wildtype LS101(WT) and isogenic mutant tagged with sig70c35 lux. **B)** After infection of LS101_sig70c35 lux_ strain and LS101_sig70c35 lump.lux_ _._ Each symbol represents a bird, and the median value is indicated by horizontal bar. The dotted horizontal line indicates the detection limit. Mann-Whitney test used to calculate the statistical significancy (WT vs mutant colony counts). ns; not significant, *= 0.0159.

**A**

**B**

| Bacterial strain | Description and/or genotype | Reference |
| --- | --- | --- |
| Sal18 (Wildtype) | Salmonella enterica serovar Enteritidis wild type | [40,41] |
| LS101(Wildtype) | Salmonella enterica serovar Enteritidis wild type | [17] |
| LS183 (Wildtype) | Salmonella enterica serovar Enteritidis wild type | This study |
| LS 172 (Sal18_c35 lux.CmR_) | Sal18, *att* Tn7::sig70c35 *luxCDABE.cat* | This study |
| LS190 (WT_c35 lux.CmR_) | LS101, *att* Tn7::sig70c35 *luxCDABE.cat* | [17] |
| LS 189 (LS183_c35.CmR_) | LS183, *att* Tn7::sig70c35 *luxCDABE.cat* | This study |
| LS191(Sal18_c35lump. lux_) | Sal18, *att* Tn7:: sig70c35 *lump*. *luxCDABE.cat* | This study |
| LS193(Sal183_c35lump. lux_) | LS183*, att* Tn7:: sig70c35 *lump*. *luxCDABE.cat* | This study |
| LS195(LS101_c35lump.lux_) | LS101, *att* Tn7:: sig70c35 *luxCDABE.cat* | This study |
| LS228 (∆SPI-1) | LS101, *∆SPI-1 att Tn7::* sig70c35 *luxCDABE.cat* | This study |
| LS225 (∆SPI-2) | LS101, *∆SPI-2 att Tn7::* sig70c35 *luxCDABE.cat* | This study |
| LS218 (*∆pagN*) | LS101, *∆pagN att Tn7::* sig70c35 *luxCDABE.cat* | This study |
| LS226 (*∆fur)* | LS101, *∆fur att* Tn7*::* sig70c35 *luxCDABE.cat* | This study |
| LS227 (*∆tonB*) | LS101, *∆tonB att* Tn7*::* sig70c35 *luxCDABE.cat* | This study |
| *E. coli* DH5α | DH5α F− φ80dlacZΔM15 Δ(*lacZYA-argF*) *U169 deoR recA1 endA1 hsdR17*(rK− mK−) *phoA supE44 λ−thi-1 gyrA96 relA1* | Invitrogen |
| *E. coli* DH10B | F^–^*mcr*A Δ(*mrr*-*hsd*RMS-*mcr*BC) φ80*lac*ZΔM15 Δ*lac*X74 *rec*A1 *end*A1 *ara*D139 Δ(*ara-leu*)7697 *gal*U *gal*K λ^–^*rps*L(Str^R^) *nup*G | Invitrogen |
| *E. coli* CC118 | λ*pir* | Aaron white’s lab |
|  |  |  |
| Plasmids |  |  |
| PGEM-T |  | Promega |
| pSKB3-GBD-Lump | Expression vector for Lump. | Addgene |
| pCS26-Cm^r^ | Carry *luxCDABE* with BamHI and XhoI upstream for promoter cloning. Chloramphenicol resistant | [11] |
| pUC18R6K-mini-Tn7T | Tn7 elements with multiple cloning site. Modified to contain a PacI restriction site. | [11] |
| pKD46 | Encodes for lambda recombinases | [35] |
| pKD3 | Encodes for Cm marker flanked by FRT | [35] |
| pCP20 | Used to cure FRT flanked regions | [35] |

**Supplementary table 2: Bacterial strains and plasmid used in this study**

| **Primer name**  **Supplementary table 3: List of primers** | **Sequence (5'-3')** | **Purpose** |
| --- | --- | --- |
| Cm-check (Shivak DJ et al., 2017) | CCCCGTGGAGGTAATAATTG | To verify chromosomal insertion of Cm^R^ marker of the reporter |
| Lux-check (Shivak DJ et al., 2017) | TCAACACTTGTTTCTTTGAGG | To verify chromosomal insertion of *luxCDABE* |
| pZE05-RW (Shivak DJ et al., 2017) | CCAGCTGGCAATTCCGA | To verify promoter sequence in pCS26 plasmid. |
| pZE06- FW(Shivak DJ et al., 2017) | AATCATCACTTTCGGGAA |  |
| glmS detect-FW (Shivak DJ et al., 2017) | AACCACCCGTTCAGGCTGGCTA | To verify chromosomal insertion downstream of *glm*S gene |
| glmS detect-RW (Shivak DJ et al., 2017) | ACGTTGACCAGCCGCGTAAC |  |
| Lump BamHI-FW (this study). Shine Dalgarno sequence is underlined | AGATCTAGGAGGCCATATGGGCCTGA | Amplfication of *lump* from pSKB3-GBD-Lump |
| Lump BglII-RW (this study). Stop codons are underlined | GGATCCTACTACCATTCATTTAAAATTTCAACG |  |
|  |  |  |
| SPI-1 lamda -FW (Desin TS et al., 2009 ) | GCTGTCGCGTATGAAGCGATTGGGTATTGATAAAGACGCGTTAGCGTAA GTGTAGGCTGGAGCTGCTTC | To amplify PCR fragment containing Cm^r^ with FRT from pKD3 ,(underlined) flanked by *fhlA* and *invH* |
| SPI-1 lamda -RW(Desin TS et al.,2009) | ATATGGTCTTAATTATATCATGATGAGTTCAGCCAACGGTGATATGGCC ATATGAATATCCTCCTTAG |  |
| OL50-1 | GAA TTC TTG CGC GAA CGT CC | To verify SPI-1 replacement by Cm^r^. |
| FRT-RW (this study) | TTATACGCAAGGCGACAAGG |  |
| SPI-2 lamda -FW (Wisner ASL et al., 2010) | TCCAGGACGCGTGGTATTGGCATATCGGTGGGATGATAGCCAAGACAAAC GTGTAGGCTGGAGCTGCTTC | To amplify PCR fragment containing Cm^r^ with FRT from pKD3 ,(underlined) flanked region are *ssaU* and  *pyK* |
| SPI-2 lamda-RW (Wisner ASL et al., 2010) | TGCCTCGCTCTAAGGATAGGTGACATCGAAAGAGCGTGCAGAGGAATGTG ATATGAATATCCTCCTTAG |  |
| KO-1 -FW | TGGATACGCTGACCGTGTTC | To verify SPI-2 replacement by Cm^r^. |
| KO-4 RW | ATACCAGTTTCGCGGTACCC |  |
| PagN lamda-FW (this study) | TAAAAGGCGTAAGTAATGCCGAGCATGAAGTCATTGGAGGCAGCCTTT GTGTAGGCTGGAGCTGCTTC | To amplify PCR fragment containing Cm^r^ with FRT from pKD3 ,(underlined) flanked region are *SEN01480* and *SEN RS01490* |
| PagN-lamda- RW (this study) | ATGAAAAACTTTTTCGCAGTCTGCATCATTCCCCTTGTGGTAGCCTGG ATATGAATATCCTCCTTAG |  |
| PagN check-FW (this study) | TTAAAAGGCGTAAGTAATGCCGAGC | To verify *pagN* replacement by Cm^r^. |
| PagN check -RW (this study) | GACCGTTTTTTGGGCTTCGTTTATG |  |
| Fur lamda-FW | GAGTGCAATTTCTGTCACTTCTCTAATGAAGTGAATCGCTTAGCAACAGG GTGTAGGCTGGAGCTGCTTC | To amplify PCR fragment containing Cm^r^ with FRT from pKD3 ,(underlined) flanked regions ; *lysR* and fldA |
| Fur Lamda-RW (this study) | ATATAAAAAAGCCAACCGGGCGGTTGGCTCTTCGAAAGATTTACACTTA ATATGAATATCCTCCTTAG |  |
| Fur check -FW | GCTCTCCTGAGATGCGATAG | To verify *fur* replacement by Cm^r^. |
| Fur check-RW | CATCTGCGAGAGACTTGCGG |  |
| tonB lambda-FW (Wellawa DH et al., 2022) | ATGATTGCTATTTGCATTTAAAATTCAGCTCTGGTTTTTCAACTGAAACG ATATGAATATCCTCCTTAG | To amplify PCR fragment containing Cm^r^ with FRT from pKD3 ,(underlined) flanked region are *SENRS06715* and *ybgC* |
| tonB lamda-RW (Wellawa DH et al., 2022) | CTTACGCCGCCAGCAGGTGATGGTATATTCCTACTG GCGGCGCCAGAGATGTGTAGGCTGGAGCTGCTTC |  |
| TonB check- FW | GTGTGGTGTTTTTGCGCGAT | To verify *tonB* replacement by Cm^r^. |
| TonB check-RW | GGACGGTAAACCTCGCCCGC |  |
